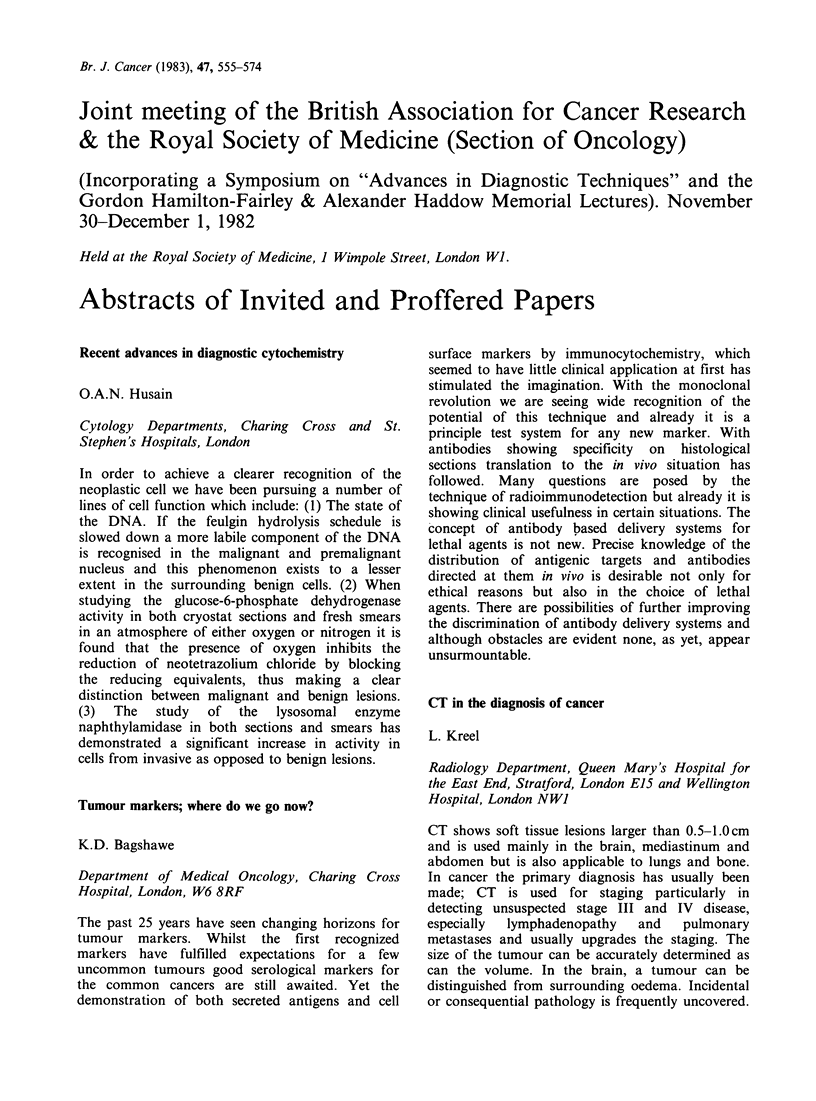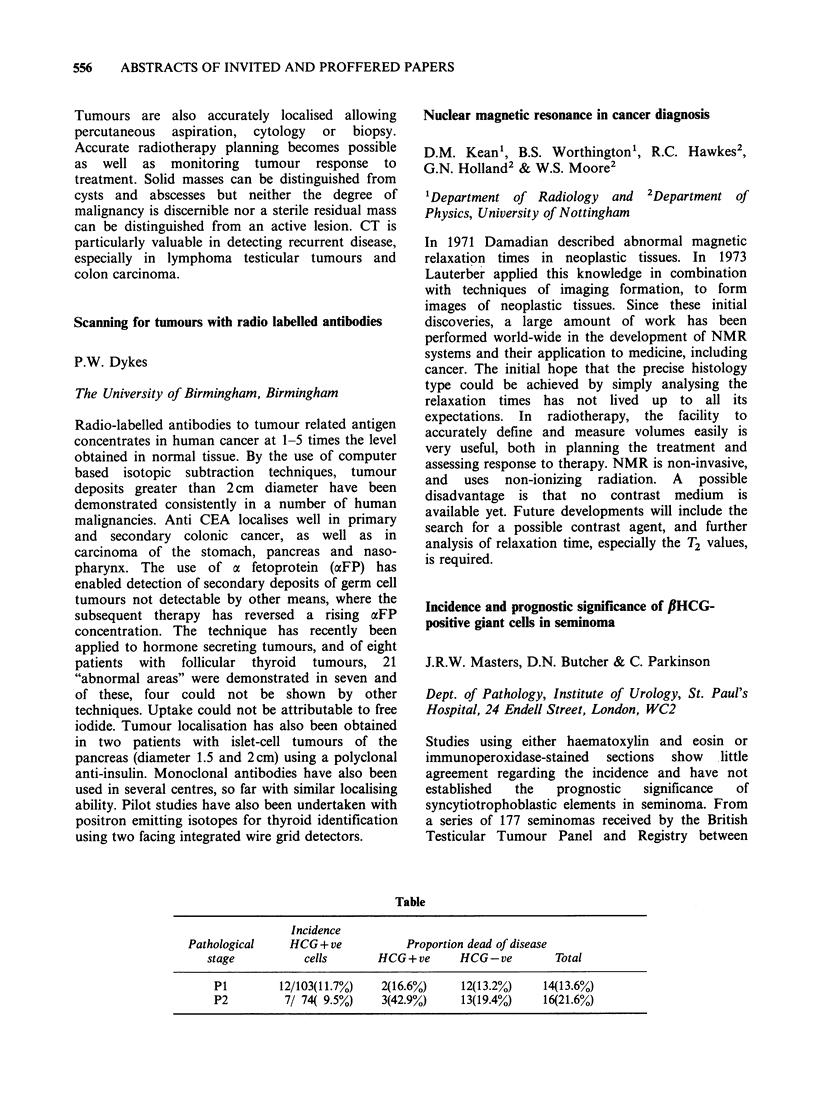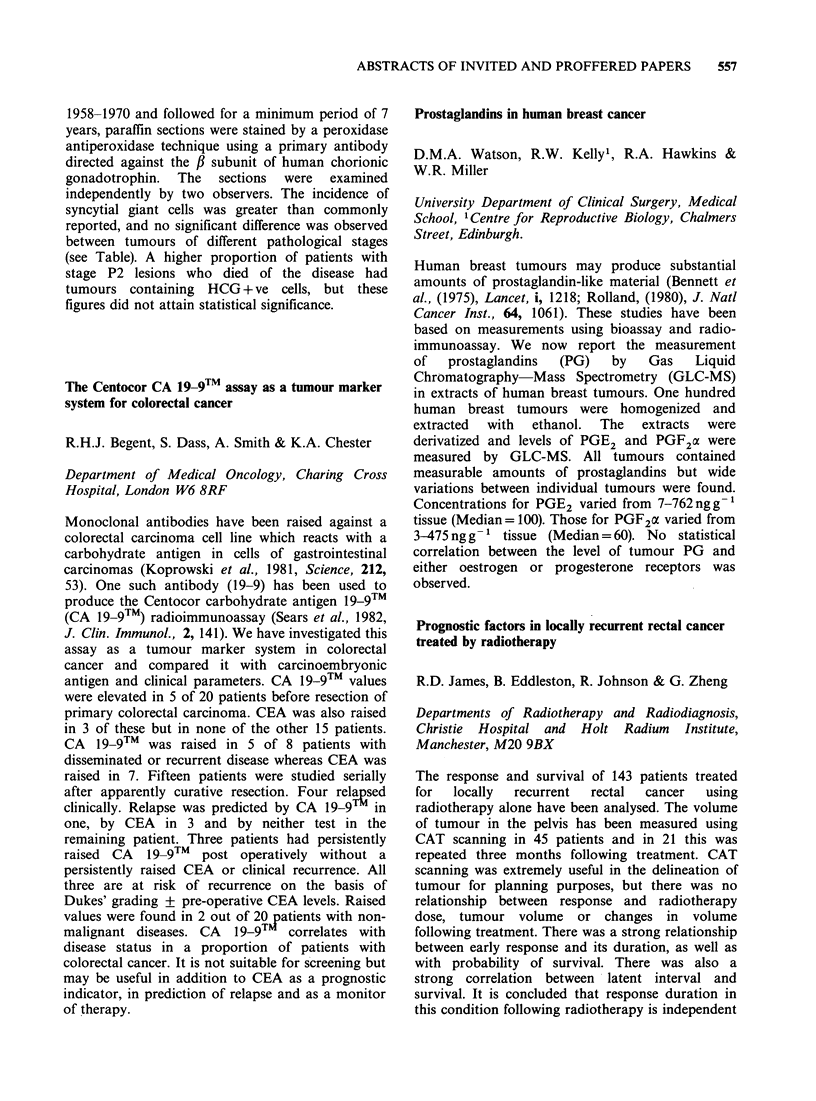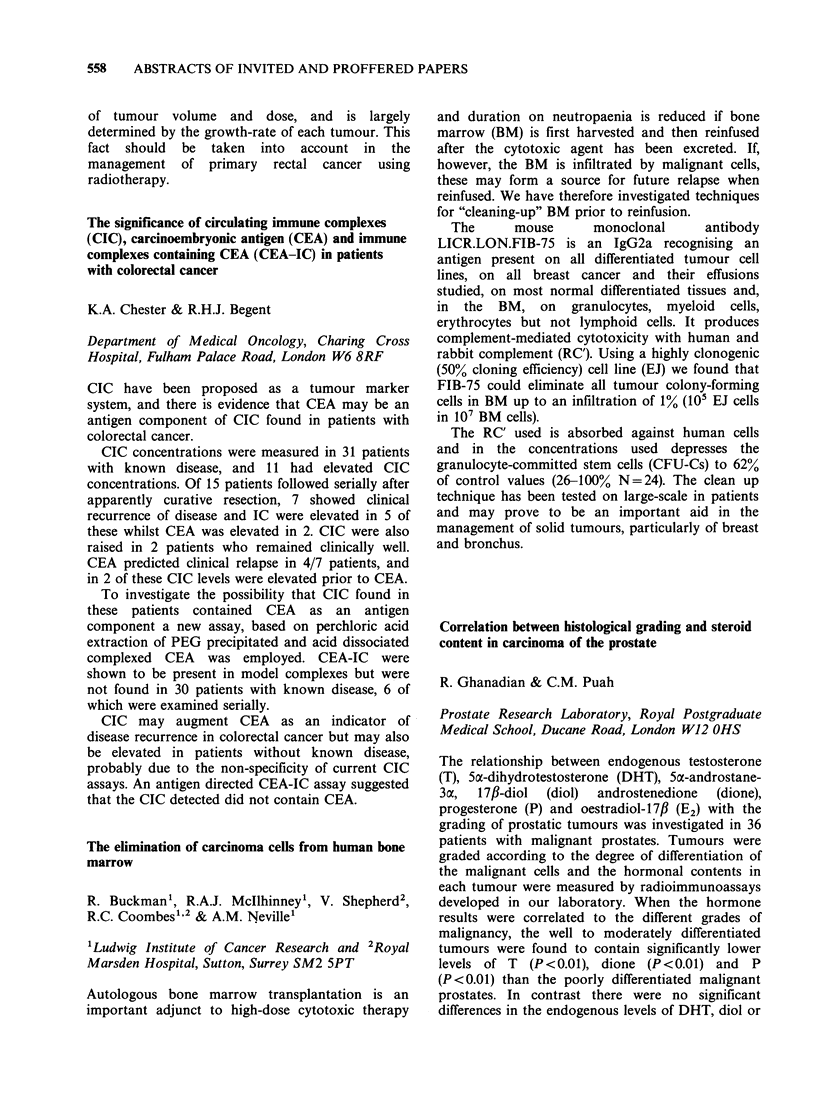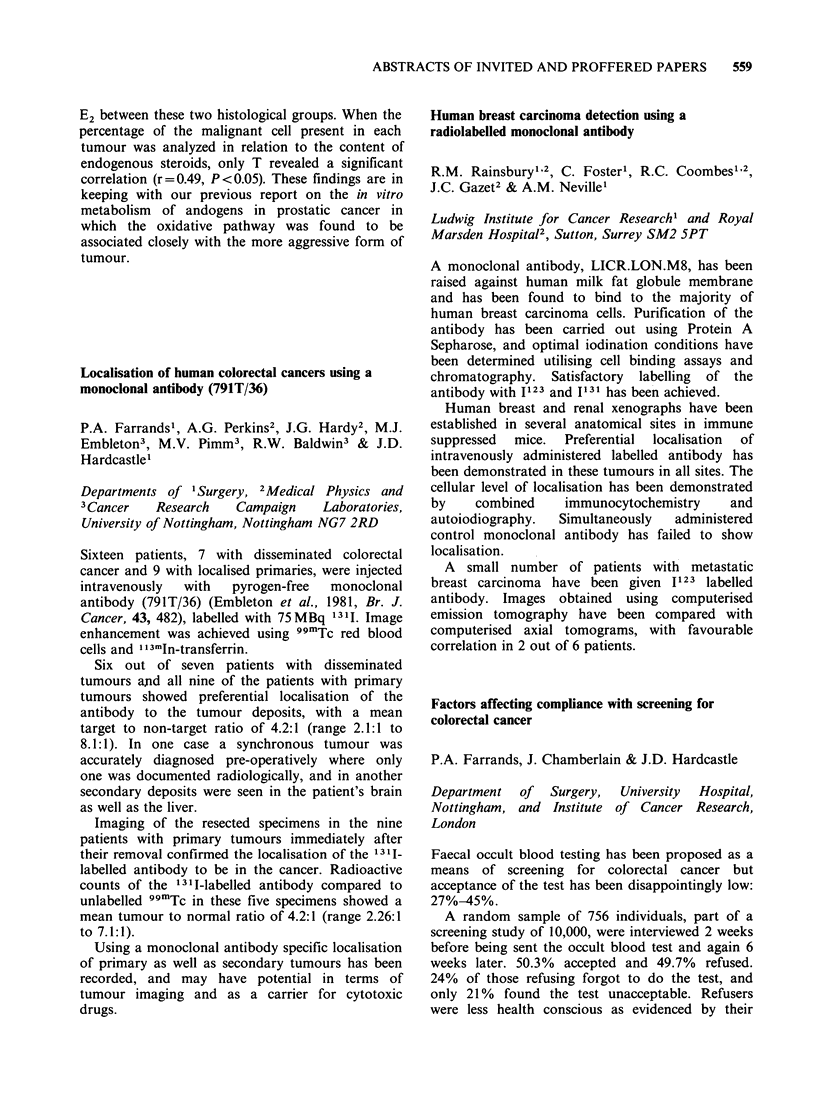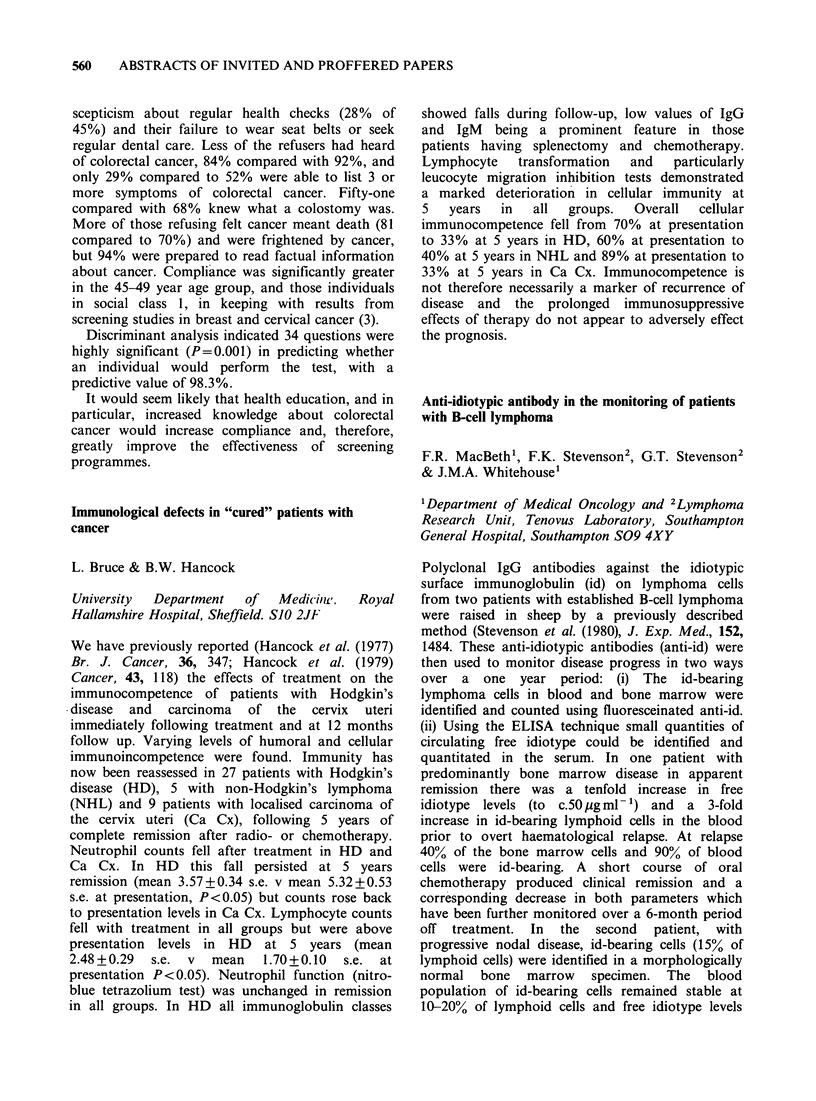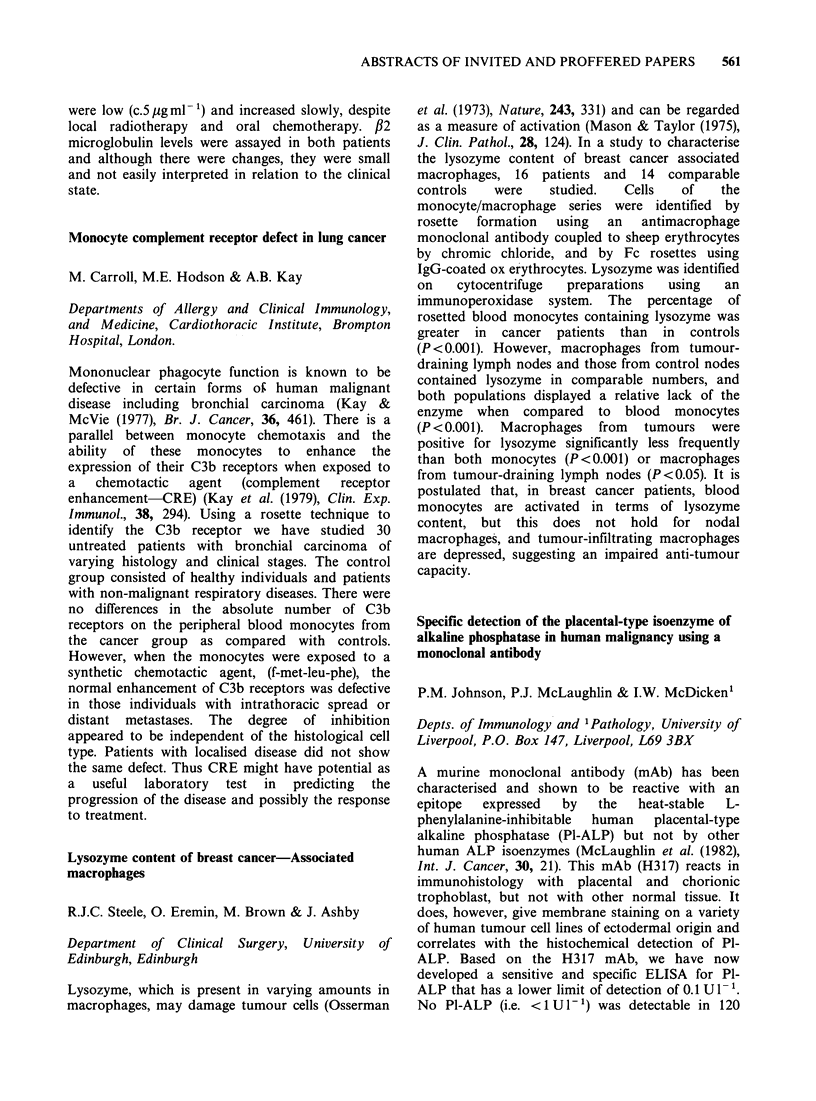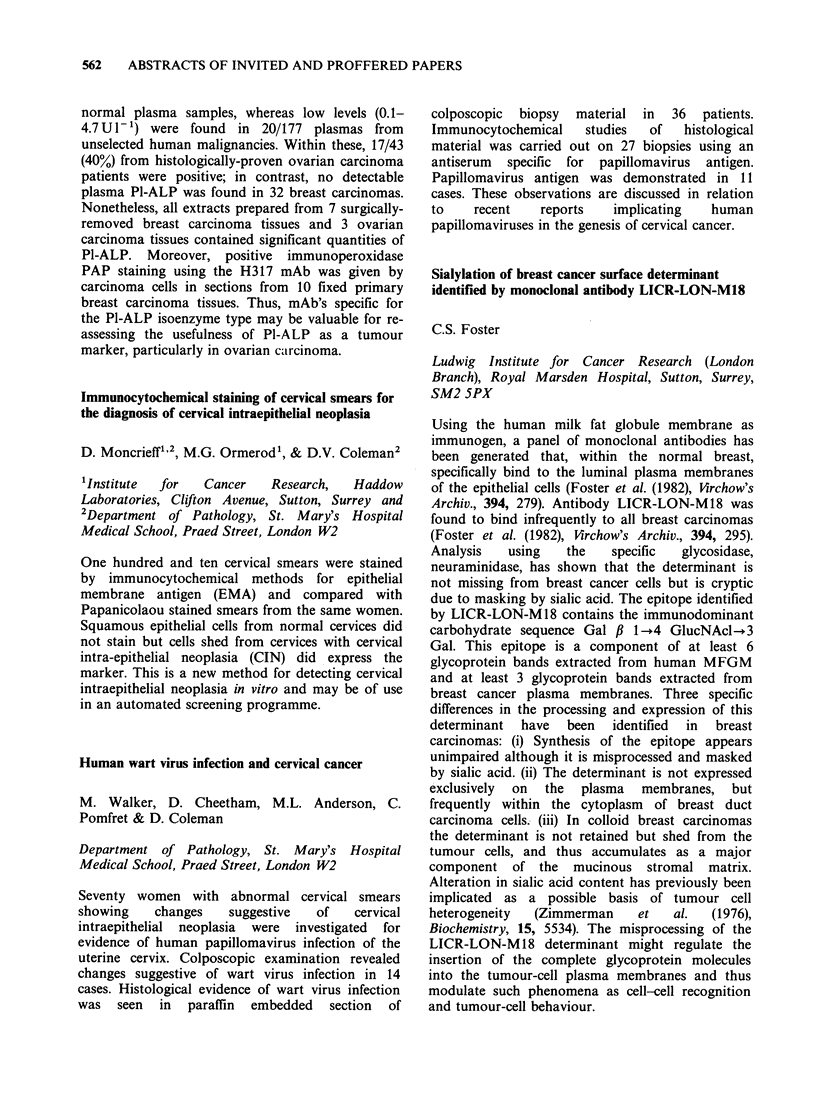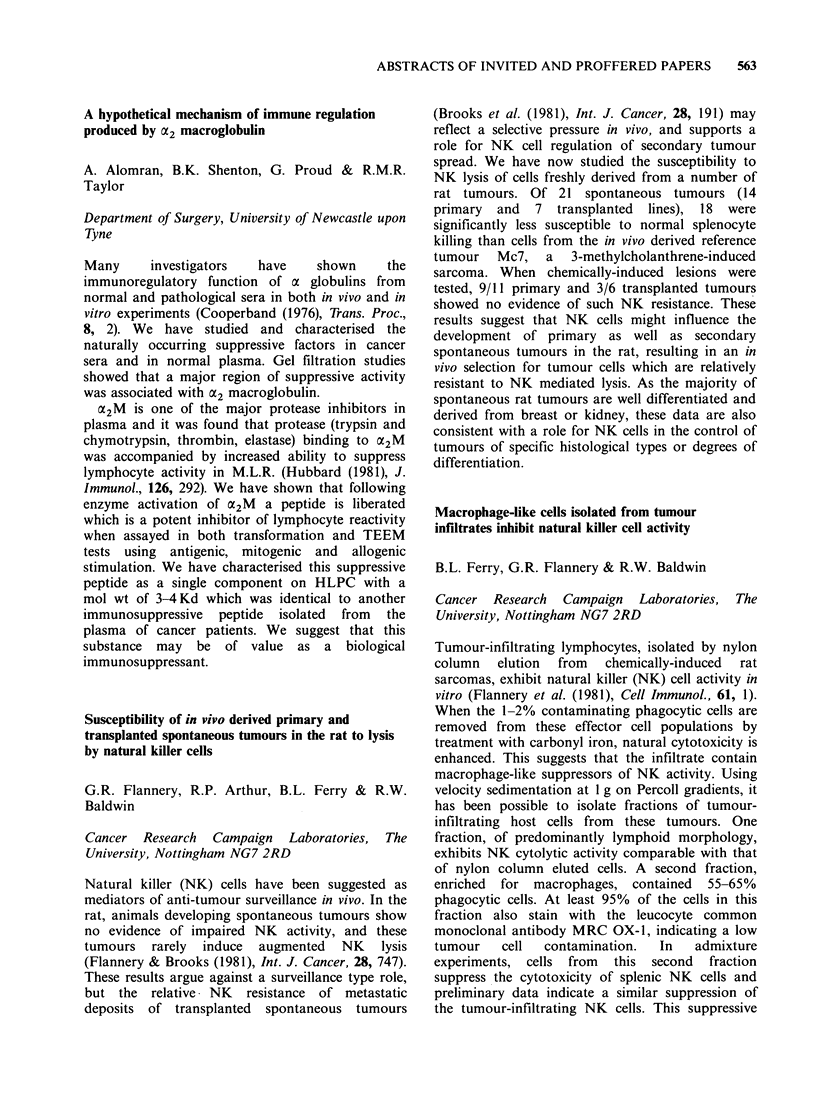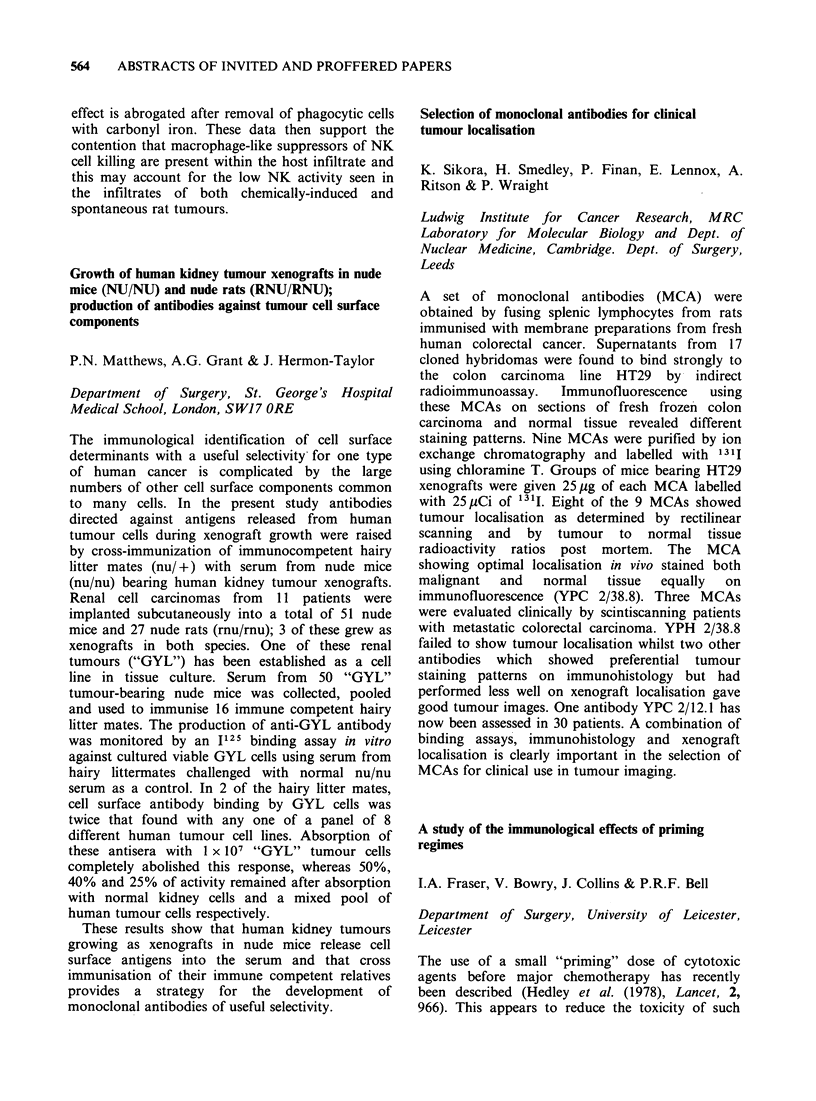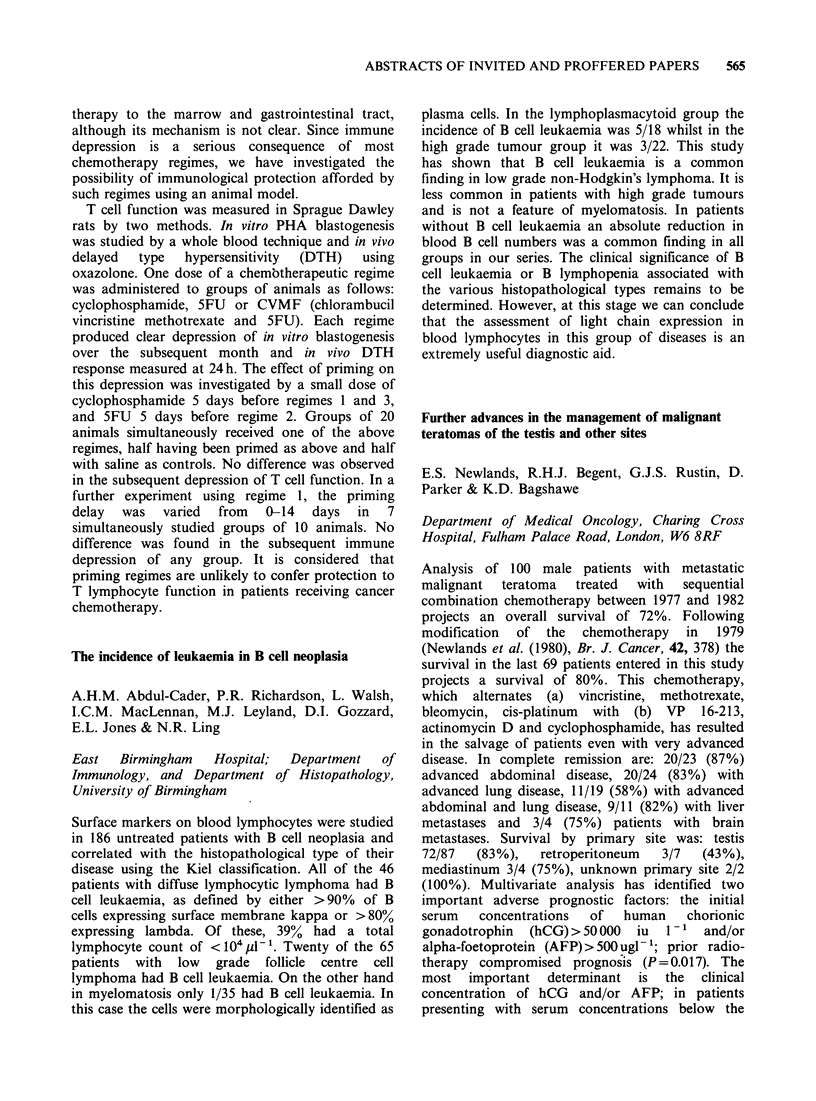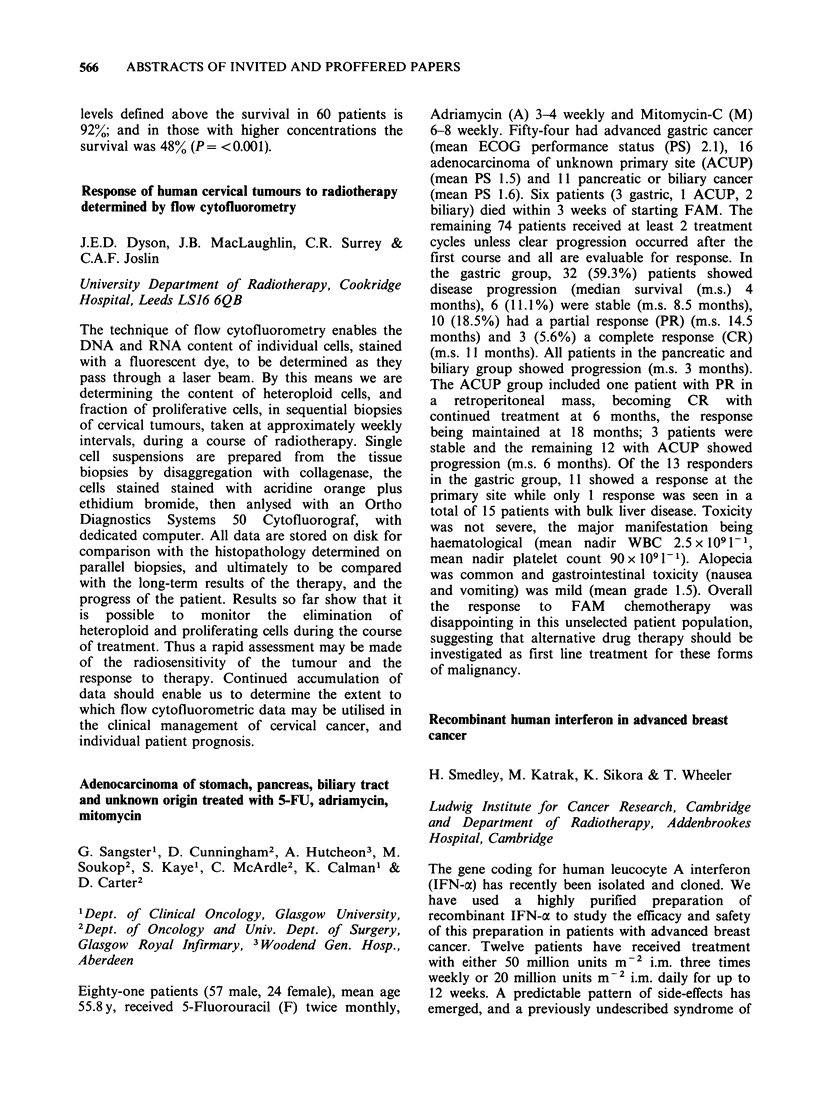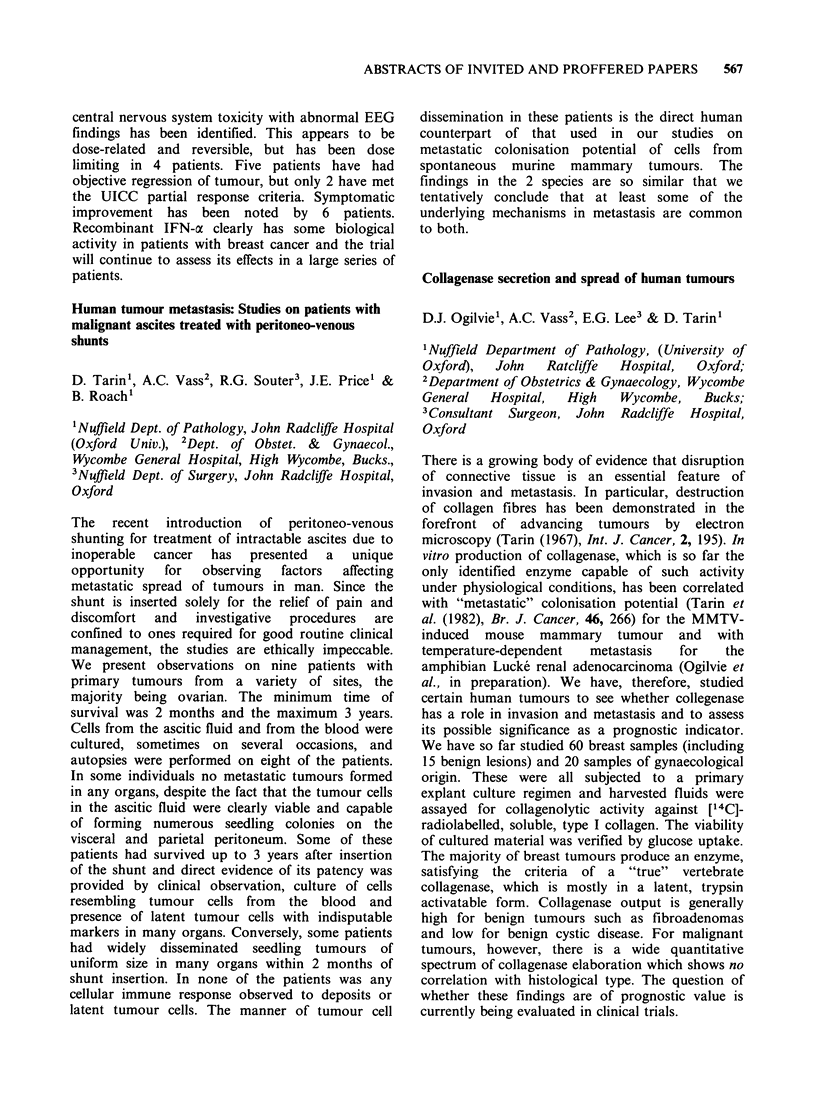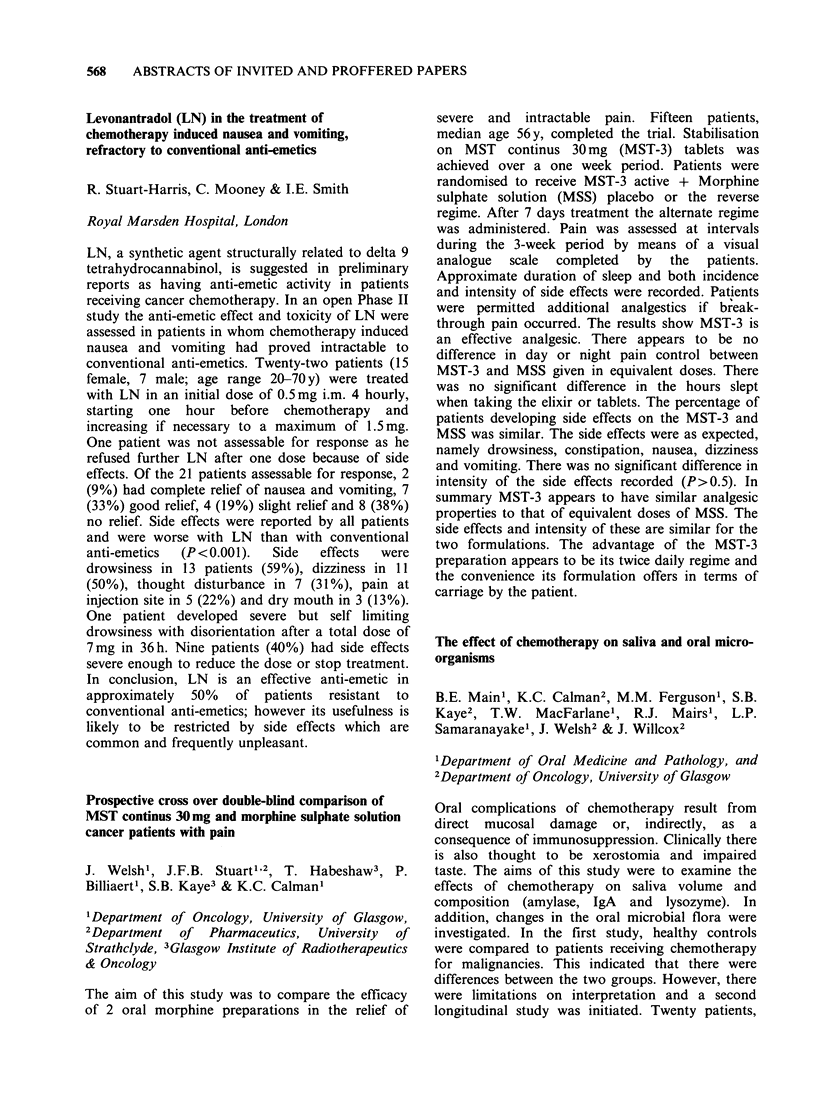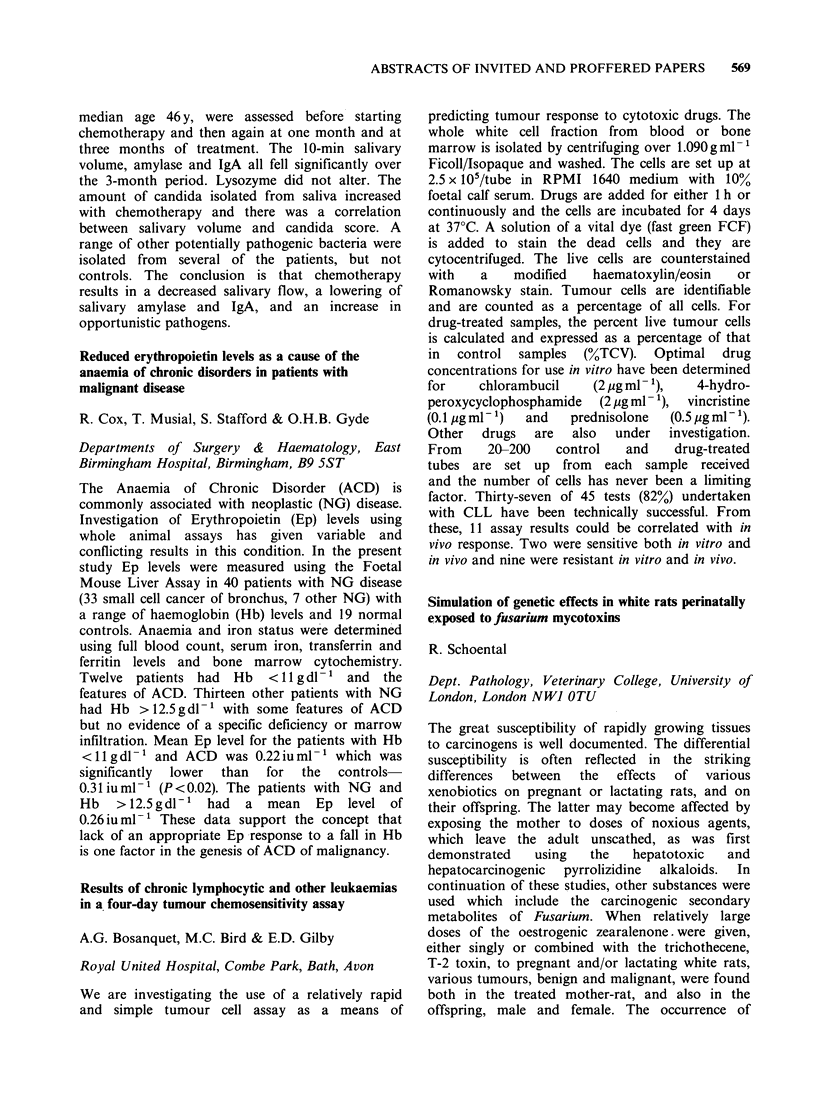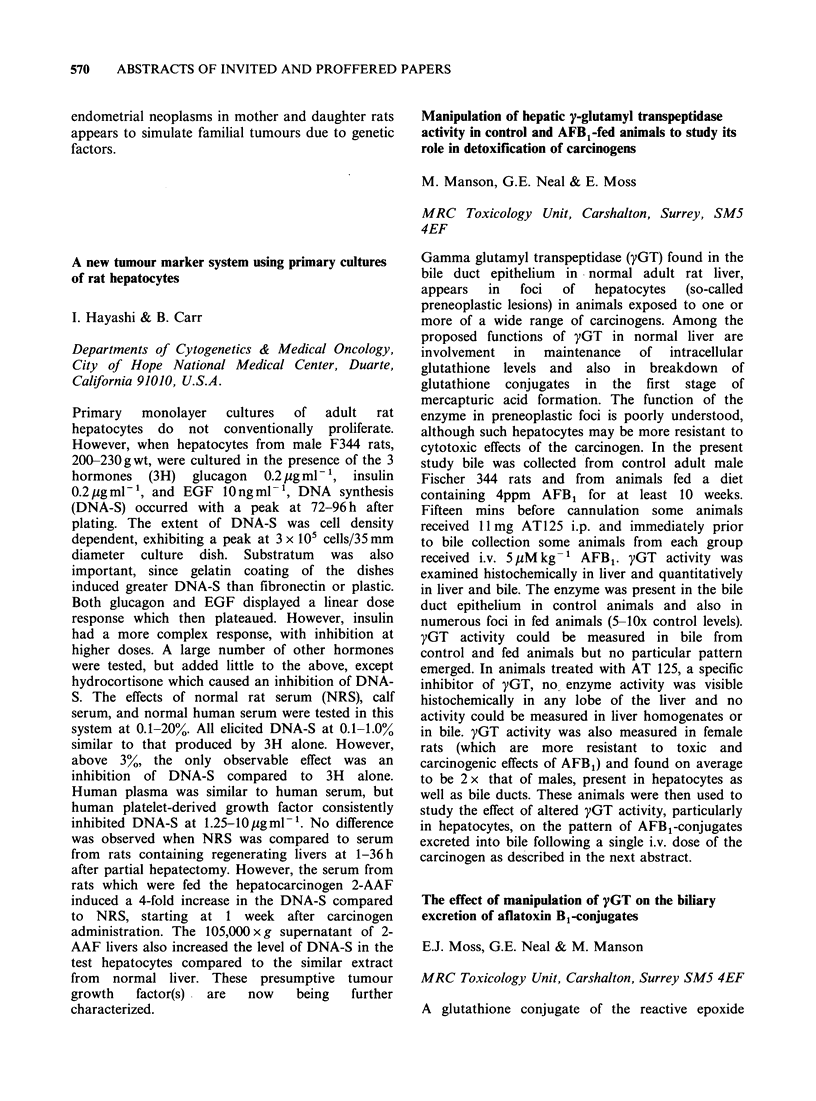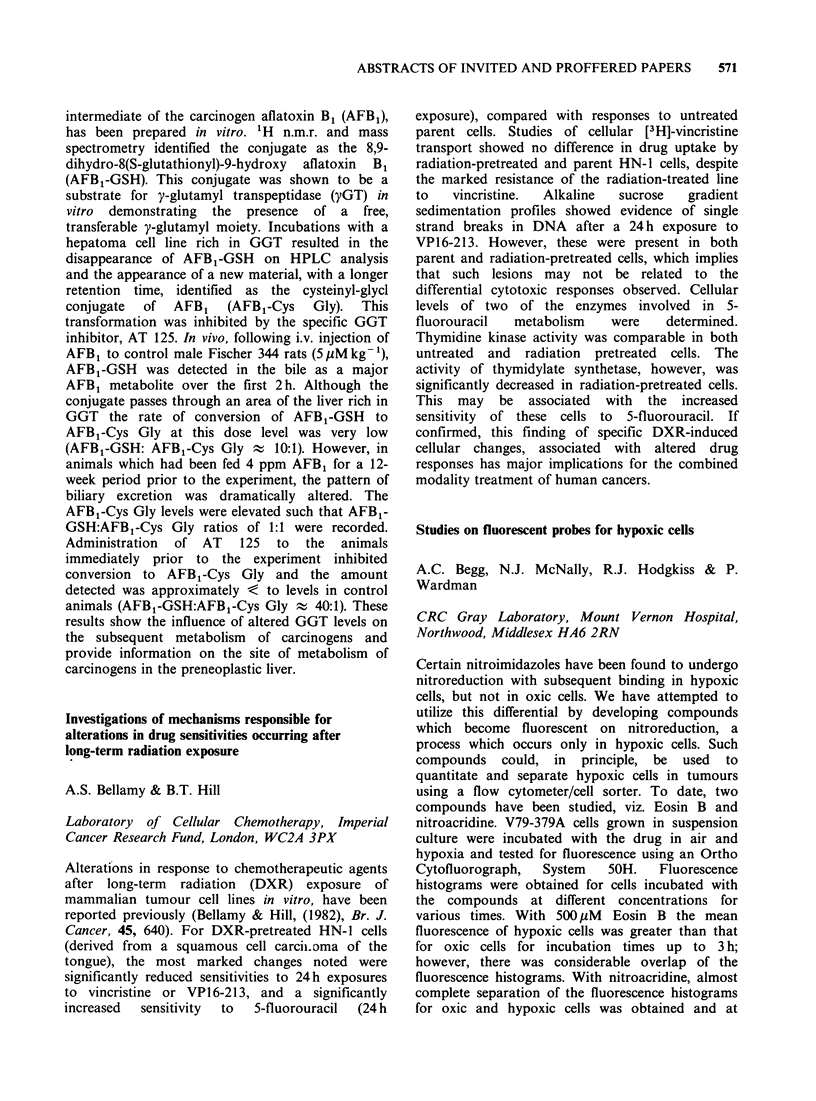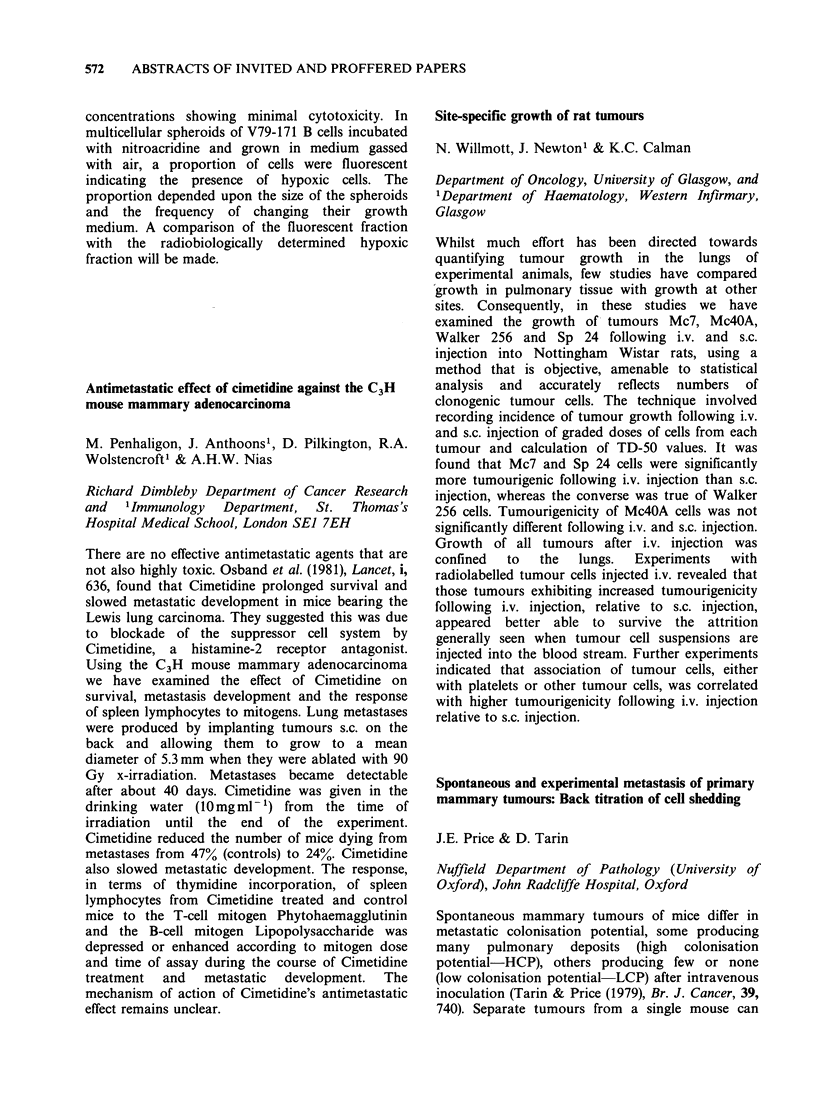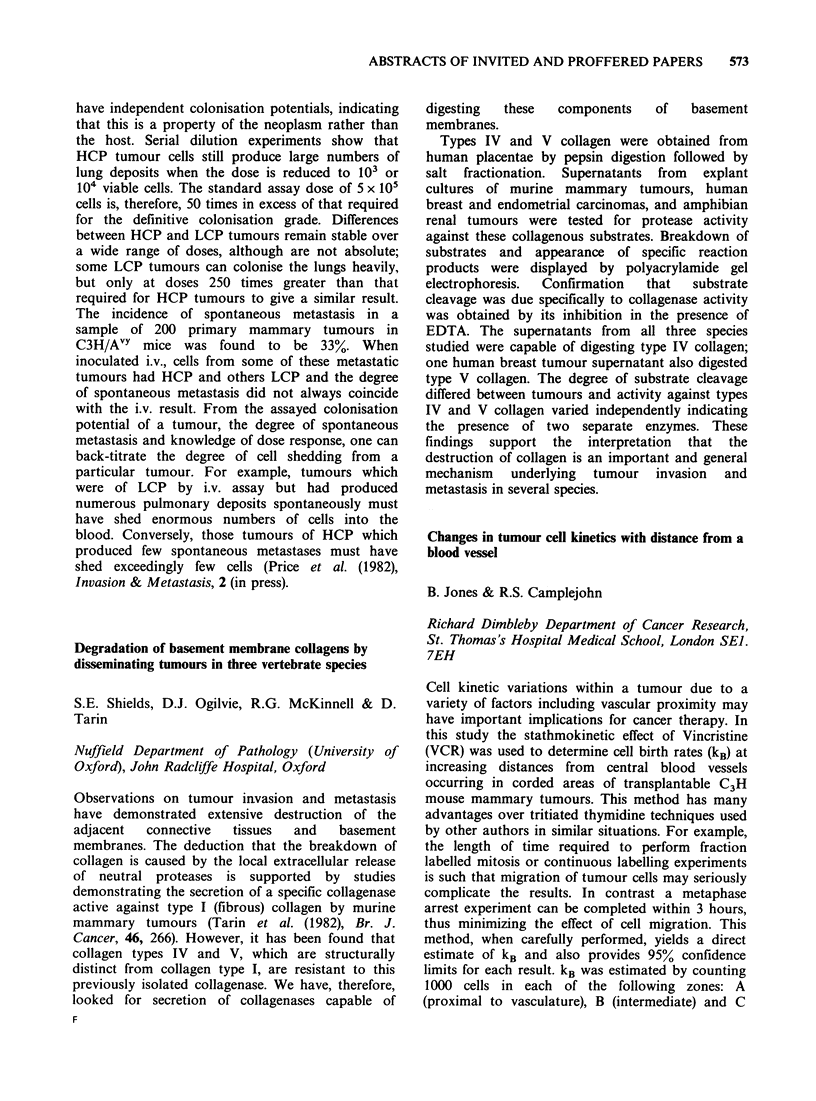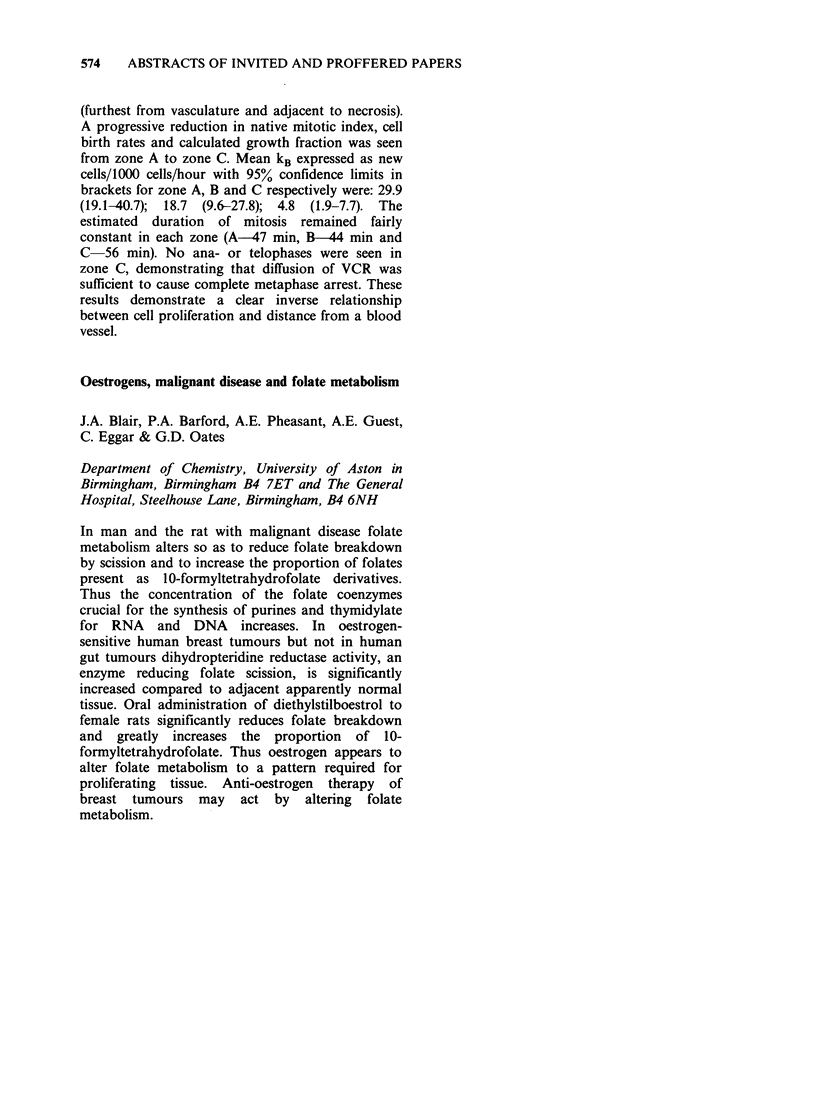# British Association for Cancer Research—Proceedings of Joint Meeting with Section of Oncology, Royal Society of Medicine, December 1982

**Published:** 1983-04

**Authors:** 


Br. J. Cancer (1983), 47, 555–574

### Joint meeting of the British Association for Cancer Research & the Royal Society of Medicine (Section of Oncology)

### (Incorporating a Symposium on "Advances in Diagnostic Techniques" and the Gordon Hamilton-Fairley & Alexander Haddow Memorial Lectures). November 30–December 1, 1982

*Held at the Royal Society of Medicine, 1 Wimpole Street, London W1.*

### Abstracts of Invited and Proffered Papers

### Recent advances in diagnostic cytochemistry

O.A.N. Husain

*Cytology Departments, Charing Cross and St. Stephen's Hospitals, London*

In order to achieve a clearer recognition of the neoplastic cell we have been pursuing a number of lines of cell function which include: (1) The state of the DNA. If the feulgin hydrolysis schedule is slowed down a more labile component of the DNA is recognised in the malignant and premalignant nucleus and this phenomenon exists to a lesser extent in the surrounding benign cells. (2) When studying the glucose-6-phosphate dehydrogenase activity in both cryostat sections and fresh smears in an atmosphere of either oxygen or nitrogen it is found that the presence of oxygen inhibits the reduction of neotetrazolium chloride by blocking the reducing equivalents, thus making a clear distinction between malignant and benign lesions. (3) The study of the lysosomal enzyme naphthylamidase in both sections and smears has demonstrated a significant increase in activity in cells from invasive as opposed to benign lesions.

### Tumour markers; where do we go now?

K.D. Bagshawe

*Department of Medical Oncology, Charing Cross Hospital, London, W6 8RF*

The past 25 years have seen changing horizons for tumour markers. Whilst the first recognized markers have fulfilled expectations for a few uncommon tumours good serological markers for the common cancers are still awaited. Yet the demonstration of both secreted antigens and cell surface markers by immunocytochemistry, which seemed to have little clinical application at first has stimulated the imagination. With the monoclonal revolution we are seeing wide recognition of the potential of this technique and already it is a principle test system for any new marker. With antibodies showing specificity on histological sections translation to the *in vivo* situation has followed. Many questions are posed by the technique of radioimmunodetection but already it is showing clinical usefulness in certain situations. The concept of antibody based delivery systems for lethal agents is not new. Precise knowledge of the distribution of antigenic targets and antibodies directed at them *in vivo* is desirable not only for ethical reasons but also in the choice of lethal agents. There are possibilities of further improving the discrimination of antibody delivery systems and although obstacles are evident none, as yet, appear unsurmountable.

### CT in the diagnosis of cancer

L. Kreel

*Radiology Department, Queen Mary's Hospital for the East End, Stratford, London E15 and Wellington Hospital, London NW1*

CT shows soft tissue lesions larger than 0.5–1.0 cm and is used mainly in the brain, mediastinum and abdomen but is also applicable to lungs and bone. In cancer the primary diagnosis has usually been made; CT is used for staging particularly in detecting unsuspected stage III and IV disease, especially lymphadenopathy and pulmonary metastases and usually upgrades the staging. The size of the tumour can be accurately determined as can the volume. In the brain, a tumour can be distinguished from surrounding oedema. Incidental or consequential pathology is frequently uncovered.

Tumours are also accurately localised allowing percutaneous aspiration, cytology or biopsy. Accurate radiotherapy planning becomes possible as well as monitoring tumour response to treatment. Solid masses can be distinguished from cysts and abscesses but neither the degree of malignancy is discernible nor a sterile residual mass can be distinguished from an active lesion. CT is particularly valuable in detecting recurrent disease, especially in lymphoma testicular tumours and colon carcinoma.

### Scanning for tumours with radio labelled antibodies

P.W. Dykes

*The University of Birmingham, Birmingham*

Radio-labelled antibodies to tumour related antigen concentrates in human cancer at 1–5 times the level obtained in normal tissue. By the use of computer based isotopic subtraction techniques, tumour deposits greater than 2 cm diameter have been demonstrated consistently in a number of human malignancies. Anti CEA localises well in primary and secondary colonic cancer, as well as in carcinoma of the stomach, pancreas and naso-pharynx. The use of α fetoprotein (αFP) has enabled detection of secondary deposits of germ cell tumours not detectable by other means, where the subsequent therapy has reversed a rising αFP concentration. The technique has recently been applied to hormone secreting tumours, and of eight patients with follicular thyroid tumours, 21 "abnormal areas" were demonstrated in seven and of these, four could not be shown by other techniques. Uptake could not be attributable to free iodide. Tumour localisation has also been obtained in two patients with islet-cell tumours of the pancreas (diameter 1.5 and 2 cm) using a polyclonal anti-insulin. Monoclonal antibodies have also been used in several centres, so far with similar localising ability. Pilot studies have also been undertaken with positron emitting isotopes for thyroid identification using two facing integrated wire grid detectors.

### Nuclear magnetic resonance in cancer diagnosis

D.M. Kean[1], B.S. Worthington[1], R.C. Hawkes[2], G.N. Holland[2] & W.S. Moore[2]

[1]*Department of Radiology and* [2]*Department of Physics, University of Nottingham*

In 1971 Damadian described abnormal magnetic relaxation times in neoplastic tissues. In 1973 Lauterber applied this knowledge in combination with techniques of imaging formation, to form images of neoplastic tissues. Since these initial discoveries, a large amount of work has been performed world-wide in the development of NMR systems and their application to medicine, including cancer. The initial hope that the precise histology type could be achieved by simply analysing the relaxation times has not lived up to all its expectations. In radiotherapy, the facility to accurately define and measure volumes easily is very useful, both in planning the treatment and assessing response to therapy. NMR is non-invasive, and uses non-ionizing radiation. A possible disadvantage is that no contrast medium is available yet. Future developments will include the search for a possible contrast agent, and further analysis of relaxation time, especially the $T_2$ values, is required.

### Incidence and prognostic significance of βHCG-positive giant cells in seminoma

J.R.W. Masters, D.N. Butcher & C. Parkinson

*Dept. of Pathology, Institute of Urology, St. Paul's Hospital, 24 Endell Street, London, WC2*

Studies using either haematoxylin and eosin or immunoperoxidase-stained sections show little agreement regarding the incidence and have not established the prognostic significance of syncytiotrophoblastic elements in seminoma. From a series of 177 seminomas received by the British Testicular Tumour Panel and Registry between

**Table**

| *Pathological stage* | *Incidence HCG+ve cells* | *Proportion dead of disease* | | |
|---|---|---|---|---|
| | | *HCG+ve* | *HCG−ve* | *Total* |
| P1 | 12/103(11.7%) | 2(16.6%) | 12(13.2%) | 14(13.6%) |
| P2 | 7/ 74( 9.5%) | 3(42.9%) | 13(19.4%) | 16(21.6%) |

1958–1970 and followed for a minimum period of 7 years, paraffin sections were stained by a peroxidase antiperoxidase technique using a primary antibody directed against the $\beta$ subunit of human chorionic gonadotrophin. The sections were examined independently by two observers. The incidence of syncytial giant cells was greater than commonly reported, and no significant difference was observed between tumours of different pathological stages (see Table). A higher proportion of patients with stage P2 lesions who died of the disease had tumours containing HCG+ve cells, but these figures did not attain statistical significance.

### The Centocor CA 19–9$^{TM}$ assay as a tumour marker system for colorectal cancer

R.H.J. Begent, S. Dass, A. Smith & K.A. Chester

*Department of Medical Oncology, Charing Cross Hospital, London W6 8RF*

Monoclonal antibodies have been raised against a colorectal carcinoma cell line which reacts with a carbohydrate antigen in cells of gastrointestinal carcinomas (Koprowski *et al.*, 1981, *Science,* **212,** 53). One such antibody (19–9) has been used to produce the Centocor carbohydrate antigen 19–9$^{TM}$ (CA 19–9$^{TM}$) radioimmunoassay (Sears *et al.*, 1982, *J. Clin. Immunol.*, **2,** 141). We have investigated this assay as a tumour marker system in colorectal cancer and compared it with carcinoembryonic antigen and clinical parameters. CA 19–9$^{TM}$ values were elevated in 5 of 20 patients before resection of primary colorectal carcinoma. CEA was also raised in 3 of these but in none of the other 15 patients. CA 19–9$^{TM}$ was raised in 5 of 8 patients with disseminated or recurrent disease whereas CEA was raised in 7. Fifteen patients were studied serially after apparently curative resection. Four relapsed clinically. Relapse was predicted by CA 19–9$^{TM}$ in one, by CEA in 3 and by neither test in the remaining patient. Three patients had persistently raised CA 19–9$^{TM}$ post operatively without a persistently raised CEA or clinical recurrence. All three are at risk of recurrence on the basis of Dukes' grading $\pm$ pre-operative CEA levels. Raised values were found in 2 out of 20 patients with non-malignant diseases. CA 19–9$^{TM}$ correlates with disease status in a proportion of patients with colorectal cancer. It is not suitable for screening but may be useful in addition to CEA as a prognostic indicator, in prediction of relapse and as a monitor of therapy.

### Prostaglandins in human breast cancer

D.M.A. Watson, R.W. Kelly[1], R.A. Hawkins & W.R. Miller

*University Department of Clinical Surgery, Medical School, [1]Centre for Reproductive Biology, Chalmers Street, Edinburgh.*

Human breast tumours may produce substantial amounts of prostaglandin-like material (Bennett *et al.*, (1975), *Lancet,* **i,** 1218; Rolland, (1980), *J. Natl Cancer Inst.*, **64,** 1061). These studies have been based on measurements using bioassay and radioimmunoassay. We now report the measurement of prostaglandins (PG) by Gas Liquid Chromatography—Mass Spectrometry (GLC-MS) in extracts of human breast tumours. One hundred human breast tumours were homogenized and extracted with ethanol. The extracts were derivatized and levels of $PGE_2$ and $PGF_2\alpha$ were measured by GLC-MS. All tumours contained measurable amounts of prostaglandins but wide variations between individual tumours were found. Concentrations for $PGE_2$ varied from 7–762 $ng\,g^{-1}$ tissue (Median = 100). Those for $PGF_2\alpha$ varied from 3–475 $ng\,g^{-1}$ tissue (Median = 60). No statistical correlation between the level of tumour PG and either oestrogen or progesterone receptors was observed.

### Prognostic factors in locally recurrent rectal cancer treated by radiotherapy

R.D. James, B. Eddleston, R. Johnson & G. Zheng

*Departments of Radiotherapy and Radiodiagnosis, Christie Hospital and Holt Radium Institute, Manchester, M20 9BX*

The response and survival of 143 patients treated for locally recurrent rectal cancer using radiotherapy alone have been analysed. The volume of tumour in the pelvis has been measured using CAT scanning in 45 patients and in 21 this was repeated three months following treatment. CAT scanning was extremely useful in the delineation of tumour for planning purposes, but there was no relationship between response and radiotherapy dose, tumour volume or changes in volume following treatment. There was a strong relationship between early response and its duration, as well as with probability of survival. There was also a strong correlation between latent interval and survival. It is concluded that response duration in this condition following radiotherapy is independent of tumour volume and dose, and is largely determined by the growth-rate of each tumour. This fact should be taken into account in the management of primary rectal cancer using radiotherapy.

### The significance of circulating immune complexes (CIC), carcinoembryonic antigen (CEA) and immune complexes containing CEA (CEA–IC) in patients with colorectal cancer

K.A. Chester & R.H.J. Begent

*Department of Medical Oncology, Charing Cross Hospital, Fulham Palace Road, London W6 8RF*

CIC have been proposed as a tumour marker system, and there is evidence that CEA may be an antigen component of CIC found in patients with colorectal cancer.

CIC concentrations were measured in 31 patients with known disease, and 11 had elevated CIC concentrations. Of 15 patients followed serially after apparently curative resection, 7 showed clinical recurrence of disease and IC were elevated in 5 of these whilst CEA was elevated in 2. CIC were also raised in 2 patients who remained clinically well. CEA predicted clinical relapse in 4/7 patients, and in 2 of these CIC levels were elevated prior to CEA.

To investigate the possibility that CIC found in these patients contained CEA as an antigen component a new assay, based on perchloric acid extraction of PEG precipitated and acid dissociated complexed CEA was employed. CEA-IC were shown to be present in model complexes but were not found in 30 patients with known disease, 6 of which were examined serially.

CIC may augment CEA as an indicator of disease recurrence in colorectal cancer but may also be elevated in patients without known disease, probably due to the non-specificity of current CIC assays. An antigen directed CEA-IC assay suggested that the CIC detected did not contain CEA.

### The elimination of carcinoma cells from human bone marrow

R. Buckman[1], R.A.J. McIlhinney[1], V. Shepherd[2], R.C. Coombes[1,2] & A.M. Neville[1]

*[1]Ludwig Institute of Cancer Research and [2]Royal Marsden Hospital, Sutton, Surrey SM2 5PT*

Autologous bone marrow transplantation is an important adjunct to high-dose cytotoxic therapy and duration on neutropaenia is reduced if bone marrow (BM) is first harvested and then reinfused after the cytotoxic agent has been excreted. If, however, the BM is infiltrated by malignant cells, these may form a source for future relapse when reinfused. We have therefore investigated techniques for "cleaning-up" BM prior to reinfusion.

The mouse monoclonal antibody LICR.LON.FIB-75 is an IgG2a recognising an antigen present on all differentiated tumour cell lines, on all breast cancer and their effusions studied, on most normal differentiated tissues and, in the BM, on granulocytes, myeloid cells, erythrocytes but not lymphoid cells. It produces complement-mediated cytotoxicity with human and rabbit complement (RC′). Using a highly clonogenic (50% cloning efficiency) cell line (EJ) we found that FIB-75 could eliminate all tumour colony-forming cells in BM up to an infiltration of 1% ($10^5$ EJ cells in $10^7$ BM cells).

The RC′ used is absorbed against human cells and in the concentrations used depresses the granulocyte-committed stem cells (CFU-Cs) to 62% of control values (26–100% $N = 24$). The clean up technique has been tested on large-scale in patients and may prove to be an important aid in the management of solid tumours, particularly of breast and bronchus.

### Correlation between histological grading and steroid content in carcinoma of the prostate

R. Ghanadian & C.M. Puah

*Prostate Research Laboratory, Royal Postgraduate Medical School, Ducane Road, London W12 0HS*

The relationship between endogenous testosterone (T), 5α-dihydrotestosterone (DHT), 5α-androstane-3α, 17β-diol (diol) androstenedione (dione), progesterone (P) and oestradiol-17β ($E_2$) with the grading of prostatic tumours was investigated in 36 patients with malignant prostates. Tumours were graded according to the degree of differentiation of the malignant cells and the hormonal contents in each tumour were measured by radioimmunoassays developed in our laboratory. When the hormone results were correlated to the different grades of malignancy, the well to moderately differentiated tumours were found to contain significantly lower levels of T ($P < 0.01$), dione ($P < 0.01$) and P ($P < 0.01$) than the poorly differentiated malignant prostates. In contrast there were no significant differences in the endogenous levels of DHT, diol or

$E_2$ between these two histological groups. When the percentage of the malignant cell present in each tumour was analyzed in relation to the content of endogenous steroids, only T revealed a significant correlation ($r = 0.49$, $P < 0.05$). These findings are in keeping with our previous report on the *in vitro* metabolism of andogens in prostatic cancer in which the oxidative pathway was found to be associated closely with the more aggressive form of tumour.

**Localisation of human colorectal cancers using a monoclonal antibody (791T/36)**

P.A. Farrands[1], A.G. Perkins[2], J.G. Hardy[2], M.J. Embleton[3], M.V. Pimm[3], R.W. Baldwin[3] & J.D. Hardcastle[1]

*Departments of [1]Surgery, [2]Medical Physics and [3]Cancer Research Campaign Laboratories, University of Nottingham, Nottingham NG7 2RD*

Sixteen patients, 7 with disseminated colorectal cancer and 9 with localised primaries, were injected intravenously with pyrogen-free monoclonal antibody (791T/36) (Embleton *et al.*, 1981, *Br. J. Cancer*, **43**, 482), labelled with 75 MBq $^{131}$I. Image enhancement was achieved using $^{99m}$Tc red blood cells and $^{113m}$In-transferrin.

Six out of seven patients with disseminated tumours and all nine of the patients with primary tumours showed preferential localisation of the antibody to the tumour deposits, with a mean target to non-target ratio of 4.2:1 (range 2.1:1 to 8.1:1). In one case a synchronous tumour was accurately diagnosed pre-operatively where only one was documented radiologically, and in another secondary deposits were seen in the patient's brain as well as the liver.

Imaging of the resected specimens in the nine patients with primary tumours immediately after their removal confirmed the localisation of the $^{131}$I-labelled antibody to be in the cancer. Radioactive counts of the $^{131}$I-labelled antibody compared to unlabelled $^{99m}$Tc in these five specimens showed a mean tumour to normal ratio of 4.2:1 (range 2.26:1 to 7.1:1).

Using a monoclonal antibody specific localisation of primary as well as secondary tumours has been recorded, and may have potential in terms of tumour imaging and as a carrier for cytotoxic drugs.

**Human breast carcinoma detection using a radiolabelled monoclonal antibody**

R.M. Rainsbury[1,2], C. Foster[1], R.C. Coombes[1,2], J.C. Gazet[2] & A.M. Neville[1]

*Ludwig Institute for Cancer Research[1] and Royal Marsden Hospital[2], Sutton, Surrey SM2 5PT*

A monoclonal antibody, LICR.LON.M8, has been raised against human milk fat globule membrane and has been found to bind to the majority of human breast carcinoma cells. Purification of the antibody has been carried out using Protein A Sepharose, and optimal iodination conditions have been determined utilising cell binding assays and chromatography. Satisfactory labelling of the antibody with $I^{123}$ and $I^{131}$ has been achieved.

Human breast and renal xenographs have been established in several anatomical sites in immune suppressed mice. Preferential localisation of intravenously administered labelled antibody has been demonstrated in these tumours in all sites. The cellular level of localisation has been demonstrated by combined immunocytochemistry and autoiodiography. Simultaneously administered control monoclonal antibody has failed to show localisation.

A small number of patients with metastatic breast carcinoma have been given $I^{123}$ labelled antibody. Images obtained using computerised emission tomography have been compared with computerised axial tomograms, with favourable correlation in 2 out of 6 patients.

**Factors affecting compliance with screening for colorectal cancer**

P.A. Farrands, J. Chamberlain & J.D. Hardcastle

*Department of Surgery, University Hospital, Nottingham, and Institute of Cancer Research, London*

Faecal occult blood testing has been proposed as a means of screening for colorectal cancer but acceptance of the test has been disappointingly low: 27%–45%.

A random sample of 756 individuals, part of a screening study of 10,000, were interviewed 2 weeks before being sent the occult blood test and again 6 weeks later. 50.3% accepted and 49.7% refused. 24% of those refusing forgot to do the test, and only 21% found the test unacceptable. Refusers were less health conscious as evidenced by their scepticism about regular health checks (28% of 45%) and their failure to wear seat belts or seek regular dental care. Less of the refusers had heard of colorectal cancer, 84% compared with 92%, and only 29% compared to 52% were able to list 3 or more symptoms of colorectal cancer. Fifty-one compared with 68% knew what a colostomy was. More of those refusing felt cancer meant death (81 compared to 70%) and were frightened by cancer, but 94% were prepared to read factual information about cancer. Compliance was significantly greater in the 45–49 year age group, and those individuals in social class 1, in keeping with results from screening studies in breast and cervical cancer (3).

Discriminant analysis indicated 34 questions were highly significant ($P = 0.001$) in predicting whether an individual would perform the test, with a predictive value of 98.3%.

It would seem likely that health education, and in particular, increased knowledge about colorectal cancer would increase compliance and, therefore, greatly improve the effectiveness of screening programmes.

### Immunological defects in "cured" patients with cancer

L. Bruce & B.W. Hancock

*University Department of Medicine, Royal Hallamshire Hospital, Sheffield. S10 2JF*

We have previously reported (Hancock *et al.* (1977) *Br. J. Cancer,* **36,** 347; Hancock *et al.* (1979) *Cancer,* **43,** 118) the effects of treatment on the immunocompetence of patients with Hodgkin's disease and carcinoma of the cervix uteri immediately following treatment and at 12 months follow up. Varying levels of humoral and cellular immunoincompetence were found. Immunity has now been reassessed in 27 patients with Hodgkin's disease (HD), 5 with non-Hodgkin's lymphoma (NHL) and 9 patients with localised carcinoma of the cervix uteri (Ca Cx), following 5 years of complete remission after radio- or chemotherapy. Neutrophil counts fell after treatment in HD and Ca Cx. In HD this fall persisted at 5 years remission (mean $3.57 \pm 0.34$ s.e. v mean $5.32 \pm 0.53$ s.e. at presentation, $P < 0.05$) but counts rose back to presentation levels in Ca Cx. Lymphocyte counts fell with treatment in all groups but were above presentation levels in HD at 5 years (mean $2.48 \pm 0.29$ s.e. v mean $1.70 \pm 0.10$ s.e. at presentation $P < 0.05$). Neutrophil function (nitroblue tetrazolium test) was unchanged in remission in all groups. In HD all immunoglobulin classes showed falls during follow-up, low values of IgG and IgM being a prominent feature in those patients having splenectomy and chemotherapy. Lymphocyte transformation and particularly leucocyte migration inhibition tests demonstrated a marked deterioration in cellular immunity at 5 years in all groups. Overall cellular immunocompetence fell from 70% at presentation to 33% at 5 years in HD, 60% at presentation to 40% at 5 years in NHL and 89% at presentation to 33% at 5 years in Ca Cx. Immunocompetence is not therefore necessarily a marker of recurrence of disease and the prolonged immunosuppressive effects of therapy do not appear to adversely effect the prognosis.

### Anti-idiotypic antibody in the monitoring of patients with B-cell lymphoma

F.R. MacBeth[1], F.K. Stevenson[2], G.T. Stevenson[2] & J.M.A. Whitehouse[1]

[1]*Department of Medical Oncology and* [2]*Lymphoma Research Unit, Tenovus Laboratory, Southampton General Hospital, Southampton SO9 4XY*

Polyclonal IgG antibodies against the idiotypic surface immunoglobulin (id) on lymphoma cells from two patients with established B-cell lymphoma were raised in sheep by a previously described method (Stevenson *et al.* (1980), *J. Exp. Med.*, **152,** 1484. These anti-idiotypic antibodies (anti-id) were then used to monitor disease progress in two ways over a one year period: (i) The id-bearing lymphoma cells in blood and bone marrow were identified and counted using fluoresceinated anti-id. (ii) Using the ELISA technique small quantities of circulating free idiotype could be identified and quantitated in the serum. In one patient with predominantly bone marrow disease in apparent remission there was a tenfold increase in free idiotype levels (to c.50 $\mu g\,ml^{-1}$) and a 3-fold increase in id-bearing lymphoid cells in the blood prior to overt haematological relapse. At relapse 40% of the bone marrow cells and 90% of blood cells were id-bearing. A short course of oral chemotherapy produced clinical remission and a corresponding decrease in both parameters which have been further monitored over a 6-month period off treatment. In the second patient, with progressive nodal disease, id-bearing cells (15% of lymphoid cells) were identified in a morphologically normal bone marrow specimen. The blood population of id-bearing cells remained stable at 10–20% of lymphoid cells and free idiotype levels were low (c.5 $\mu g\,ml^{-1}$) and increased slowly, despite local radiotherapy and oral chemotherapy. $\beta 2$ microglobulin levels were assayed in both patients and although there were changes, they were small and not easily interpreted in relation to the clinical state.

### Monocyte complement receptor defect in lung cancer

M. Carroll, M.E. Hodson & A.B. Kay

*Departments of Allergy and Clinical Immunology, and Medicine, Cardiothoracic Institute, Brompton Hospital, London.*

Mononuclear phagocyte function is known to be defective in certain forms of human malignant disease including bronchial carcinoma (Kay & McVie (1977), *Br. J. Cancer*, **36**, 461). There is a parallel between monocyte chemotaxis and the ability of these monocytes to enhance the expression of their C3b receptors when exposed to a chemotactic agent (complement receptor enhancement—CRE) (Kay *et al.* (1979), *Clin. Exp. Immunol.*, **38**, 294). Using a rosette technique to identify the C3b receptor we have studied 30 untreated patients with bronchial carcinoma of varying histology and clinical stages. The control group consisted of healthy individuals and patients with non-malignant respiratory diseases. There were no differences in the absolute number of C3b receptors on the peripheral blood monocytes from the cancer group as compared with controls. However, when the monocytes were exposed to a synthetic chemotactic agent, (f-met-leu-phe), the normal enhancement of C3b receptors was defective in those individuals with intrathoracic spread or distant metastases. The degree of inhibition appeared to be independent of the histological cell type. Patients with localised disease did not show the same defect. Thus CRE might have potential as a useful laboratory test in predicting the progression of the disease and possibly the response to treatment.

### Lysozyme content of breast cancer—Associated macrophages

R.J.C. Steele, O. Eremin, M. Brown & J. Ashby

*Department of Clinical Surgery, University of Edinburgh, Edinburgh*

Lysozyme, which is present in varying amounts in macrophages, may damage tumour cells (Osserman *et al.* (1973), *Nature*, **243**, 331) and can be regarded as a measure of activation (Mason & Taylor (1975), *J. Clin. Pathol.*, **28**, 124). In a study to characterise the lysozyme content of breast cancer associated macrophages, 16 patients and 14 comparable controls were studied. Cells of the monocyte/macrophage series were identified by rosette formation using an antimacrophage monoclonal antibody coupled to sheep erythrocytes by chromic chloride, and by Fc rosettes using IgG-coated ox erythrocytes. Lysozyme was identified on cytocentrifuge preparations using an immunoperoxidase system. The percentage of rosetted blood monocytes containing lysozyme was greater in cancer patients than in controls ($P<0.001$). However, macrophages from tumour-draining lymph nodes and those from control nodes contained lysozyme in comparable numbers, and both populations displayed a relative lack of the enzyme when compared to blood monocytes ($P<0.001$). Macrophages from tumours were positive for lysozyme significantly less frequently than both monocytes ($P<0.001$) or macrophages from tumour-draining lymph nodes ($P<0.05$). It is postulated that, in breast cancer patients, blood monocytes are activated in terms of lysozyme content, but this does not hold for nodal macrophages, and tumour-infiltrating macrophages are depressed, suggesting an impaired anti-tumour capacity.

### Specific detection of the placental-type isoenzyme of alkaline phosphatase in human malignancy using a monoclonal antibody

P.M. Johnson, P.J. McLaughlin & I.W. McDicken[1]

*Depts. of Immunology and [1]Pathology, University of Liverpool, P.O. Box 147, Liverpool, L69 3BX*

A murine monoclonal antibody (mAb) has been characterised and shown to be reactive with an epitope expressed by the heat-stable L-phenylalanine-inhibitable human placental-type alkaline phosphatase (Pl-ALP) but not by other human ALP isoenzymes (McLaughlin *et al.* (1982), *Int. J. Cancer*, **30**, 21). This mAb (H317) reacts in immunohistology with placental and chorionic trophoblast, but not with other normal tissue. It does, however, give membrane staining on a variety of human tumour cell lines of ectodermal origin and correlates with the histochemical detection of Pl-ALP. Based on the H317 mAb, we have now developed a sensitive and specific ELISA for Pl-ALP that has a lower limit of detection of $0.1\,U\,l^{-1}$. No Pl-ALP (i.e. $<1\,U\,l^{-1}$) was detectable in 120

normal plasma samples, whereas low levels (0.1–4.7 $U l^{-1}$) were found in 20/177 plasmas from unselected human malignancies. Within these, 17/43 (40%) from histologically-proven ovarian carcinoma patients were positive; in contrast, no detectable plasma Pl-ALP was found in 32 breast carcinomas. Nonetheless, all extracts prepared from 7 surgically-removed breast carcinoma tissues and 3 ovarian carcinoma tissues contained significant quantities of Pl-ALP. Moreover, positive immunoperoxidase PAP staining using the H317 mAb was given by carcinoma cells in sections from 10 fixed primary breast carcinoma tissues. Thus, mAb's specific for the Pl-ALP isoenzyme type may be valuable for re-assessing the usefulness of Pl-ALP as a tumour marker, particularly in ovarian carcinoma.

### Immunocytochemical staining of cervical smears for the diagnosis of cervical intraepithelial neoplasia

D. Moncrieff[1,2], M.G. Ormerod[1], & D.V. Coleman[2]

[1]*Institute for Cancer Research, Haddow Laboratories, Clifton Avenue, Sutton, Surrey and* [2]*Department of Pathology, St. Mary's Hospital Medical School, Praed Street, London W2*

One hundred and ten cervical smears were stained by immunocytochemical methods for epithelial membrane antigen (EMA) and compared with Papanicolaou stained smears from the same women. Squamous epithelial cells from normal cervices did not stain but cells shed from cervices with cervical intra-epithelial neoplasia (CIN) did express the marker. This is a new method for detecting cervical intraepithelial neoplasia *in vitro* and may be of use in an automated screening programme.

### Human wart virus infection and cervical cancer

M. Walker, D. Cheetham, M.L. Anderson, C. Pomfret & D. Coleman

*Department of Pathology, St. Mary's Hospital Medical School, Praed Street, London W2*

Seventy women with abnormal cervical smears showing changes suggestive of cervical intraepithelial neoplasia were investigated for evidence of human papillomavirus infection of the uterine cervix. Colposcopic examination revealed changes suggestive of wart virus infection in 14 cases. Histological evidence of wart virus infection was seen in paraffin embedded section of colposcopic biopsy material in 36 patients. Immunocytochemical studies of histological material was carried out on 27 biopsies using an antiserum specific for papillomavirus antigen. Papillomavirus antigen was demonstrated in 11 cases. These observations are discussed in relation to recent reports implicating human papillomaviruses in the genesis of cervical cancer.

### Sialylation of breast cancer surface determinant identified by monoclonal antibody LICR-LON-M18

C.S. Foster

*Ludwig Institute for Cancer Research (London Branch), Royal Marsden Hospital, Sutton, Surrey, SM2 5PX*

Using the human milk fat globule membrane as immunogen, a panel of monoclonal antibodies has been generated that, within the normal breast, specifically bind to the luminal plasma membranes of the epithelial cells (Foster *et al.* (1982), *Virchow's Archiv.*, **394**, 279). Antibody LICR-LON-M18 was found to bind infrequently to all breast carcinomas (Foster *et al.* (1982), *Virchow's Archiv.*, **394**, 295). Analysis using the specific glycosidase, neuraminidase, has shown that the determinant is not missing from breast cancer cells but is cryptic due to masking by sialic acid. The epitope identified by LICR-LON-M18 contains the immunodominant carbohydrate sequence Gal $\beta$ 1→4 GlucNAcl→3 Gal. This epitope is a component of at least 6 glycoprotein bands extracted from human MFGM and at least 3 glycoprotein bands extracted from breast cancer plasma membranes. Three specific differences in the processing and expression of this determinant have been identified in breast carcinomas: (i) Synthesis of the epitope appears unimpaired although it is misprocessed and masked by sialic acid. (ii) The determinant is not expressed exclusively on the plasma membranes, but frequently within the cytoplasm of breast duct carcinoma cells. (iii) In colloid breast carcinomas the determinant is not retained but shed from the tumour cells, and thus accumulates as a major component of the mucinous stromal matrix. Alteration in sialic acid content has previously been implicated as a possible basis of tumour cell heterogeneity (Zimmerman *et al.* (1976), *Biochemistry*, **15**, 5534). The misprocessing of the LICR-LON-M18 determinant might regulate the insertion of the complete glycoprotein molecules into the tumour-cell plasma membranes and thus modulate such phenomena as cell–cell recognition and tumour-cell behaviour.

### A hypothetical mechanism of immune regulation produced by $\alpha_2$ macroglobulin

A. Alomran, B.K. Shenton, G. Proud & R.M.R. Taylor

*Department of Surgery, University of Newcastle upon Tyne*

Many investigators have shown the immunoregulatory function of $\alpha$ globulins from normal and pathological sera in both *in vivo* and *in vitro* experiments (Cooperband (1976), *Trans. Proc.*, **8**, 2). We have studied and characterised the naturally occurring suppressive factors in cancer sera and in normal plasma. Gel filtration studies showed that a major region of suppressive activity was associated with $\alpha_2$ macroglobulin.

$\alpha_2$M is one of the major protease inhibitors in plasma and it was found that protease (trypsin and chymotrypsin, thrombin, elastase) binding to $\alpha_2$M was accompanied by increased ability to suppress lymphocyte activity in M.L.R. (Hubbard (1981), *J. Immunol.*, **126**, 292). We have shown that following enzyme activation of $\alpha_2$M a peptide is liberated which is a potent inhibitor of lymphocyte reactivity when assayed in both transformation and TEEM tests using antigenic, mitogenic and allogenic stimulation. We have characterised this suppressive peptide as a single component on HLPC with a mol wt of 3–4 Kd which was identical to another immunosuppressive peptide isolated from the plasma of cancer patients. We suggest that this substance may be of value as a biological immunosuppressant.

### Susceptibility of *in vivo* derived primary and transplanted spontaneous tumours in the rat to lysis by natural killer cells

G.R. Flannery, R.P. Arthur, B.L. Ferry & R.W. Baldwin

*Cancer Research Campaign Laboratories, The University, Nottingham NG7 2RD*

Natural killer (NK) cells have been suggested as mediators of anti-tumour surveillance *in vivo*. In the rat, animals developing spontaneous tumours show no evidence of impaired NK activity, and these tumours rarely induce augmented NK lysis (Flannery & Brooks (1981), *Int. J. Cancer*, **28**, 747). These results argue against a surveillance type role, but the relative NK resistance of metastatic deposits of transplanted spontaneous tumours (Brooks *et al.* (1981), *Int. J. Cancer*, **28**, 191) may reflect a selective pressure *in vivo*, and supports a role for NK cell regulation of secondary tumour spread. We have now studied the susceptibility to NK lysis of cells freshly derived from a number of rat tumours. Of 21 spontaneous tumours (14 primary and 7 transplanted lines), 18 were significantly less susceptible to normal splenocyte killing than cells from the *in vivo* derived reference tumour Mc7, a 3-methylcholanthrene-induced sarcoma. When chemically-induced lesions were tested, 9/11 primary and 3/6 transplanted tumours showed no evidence of such NK resistance. These results suggest that NK cells might influence the development of primary as well as secondary spontaneous tumours in the rat, resulting in an *in vivo* selection for tumour cells which are relatively resistant to NK mediated lysis. As the majority of spontaneous rat tumours are well differentiated and derived from breast or kidney, these data are also consistent with a role for NK cells in the control of tumours of specific histological types or degrees of differentiation.

### Macrophage-like cells isolated from tumour infiltrates inhibit natural killer cell activity

B.L. Ferry, G.R. Flannery & R.W. Baldwin

*Cancer Research Campaign Laboratories, The University, Nottingham NG7 2RD*

Tumour-infiltrating lymphocytes, isolated by nylon column elution from chemically-induced rat sarcomas, exhibit natural killer (NK) cell activity *in vitro* (Flannery *et al.* (1981), *Cell Immunol.*, **61**, 1). When the 1–2% contaminating phagocytic cells are removed from these effector cell populations by treatment with carbonyl iron, natural cytotoxicity is enhanced. This suggests that the infiltrate contain macrophage-like suppressors of NK activity. Using velocity sedimentation at 1 g on Percoll gradients, it has been possible to isolate fractions of tumour-infiltrating host cells from these tumours. One fraction, of predominantly lymphoid morphology, exhibits NK cytolytic activity comparable with that of nylon column eluted cells. A second fraction, enriched for macrophages, contained 55–65% phagocytic cells. At least 95% of the cells in this fraction also stain with the leucocyte common monoclonal antibody MRC OX-1, indicating a low tumour cell contamination. In admixture experiments, cells from this second fraction suppress the cytotoxicity of splenic NK cells and preliminary data indicate a similar suppression of the tumour-infiltrating NK cells. This suppressive effect is abrogated after removal of phagocytic cells with carbonyl iron. These data then support the contention that macrophage-like suppressors of NK cell killing are present within the host infiltrate and this may account for the low NK activity seen in the infiltrates of both chemically-induced and spontaneous rat tumours.

### Growth of human kidney tumour xenografts in nude mice (NU/NU) and nude rats (RNU/RNU); production of antibodies against tumour cell surface components

P.N. Matthews, A.G. Grant & J. Hermon-Taylor

*Department of Surgery, St. George's Hospital Medical School, London, SW17 0RE*

The immunological identification of cell surface determinants with a useful selectivity for one type of human cancer is complicated by the large numbers of other cell surface components common to many cells. In the present study antibodies directed against antigens released from human tumour cells during xenograft growth were raised by cross-immunization of immunocompetent hairy litter mates (nu/+) with serum from nude mice (nu/nu) bearing human kidney tumour xenografts. Renal cell carcinomas from 11 patients were implanted subcutaneously into a total of 51 nude mice and 27 nude rats (rnu/rnu); 3 of these grew as xenografts in both species. One of these renal tumours ("GYL") has been established as a cell line in tissue culture. Serum from 50 "GYL" tumour-bearing nude mice was collected, pooled and used to immunise 16 immune competent hairy litter mates. The production of anti-GYL antibody was monitored by an $I^{125}$ binding assay *in vitro* against cultured viable GYL cells using serum from hairy littermates challenged with normal nu/nu serum as a control. In 2 of the hairy litter mates, cell surface antibody binding by GYL cells was twice that found with any one of a panel of 8 different human tumour cell lines. Absorption of these antisera with $1 \times 10^7$ "GYL" tumour cells completely abolished this response, whereas 50%, 40% and 25% of activity remained after absorption with normal kidney cells and a mixed pool of human tumour cells respectively.

These results show that human kidney tumours growing as xenografts in nude mice release cell surface antigens into the serum and that cross immunisation of their immune competent relatives provides a strategy for the development of monoclonal antibodies of useful selectivity.

### Selection of monoclonal antibodies for clinical tumour localisation

K. Sikora, H. Smedley, P. Finan, E. Lennox, A. Ritson & P. Wraight

*Ludwig Institute for Cancer Research, MRC Laboratory for Molecular Biology and Dept. of Nuclear Medicine, Cambridge. Dept. of Surgery, Leeds*

A set of monoclonal antibodies (MCA) were obtained by fusing splenic lymphocytes from rats immunised with membrane preparations from fresh human colorectal cancer. Supernatants from 17 cloned hybridomas were found to bind strongly to the colon carcinoma line HT29 by indirect radioimmunoassay. Immunofluorescence using these MCAs on sections of fresh frozen colon carcinoma and normal tissue revealed different staining patterns. Nine MCAs were purified by ion exchange chromatography and labelled with $^{131}I$ using chloramine T. Groups of mice bearing HT29 xenografts were given 25 $\mu$g of each MCA labelled with 25 $\mu$Ci of $^{131}I$. Eight of the 9 MCAs showed tumour localisation as determined by rectilinear scanning and by tumour to normal tissue radioactivity ratios post mortem. The MCA showing optimal localisation *in vivo* stained both malignant and normal tissue equally on immunofluorescence (YPC 2/38.8). Three MCAs were evaluated clinically by scintiscanning patients with metastatic colorectal carcinoma. YPH 2/38.8 failed to show tumour localisation whilst two other antibodies which showed preferential tumour staining patterns on immunohistology but had performed less well on xenograft localisation gave good tumour images. One antibody YPC 2/12.1 has now been assessed in 30 patients. A combination of binding assays, immunohistology and xenograft localisation is clearly important in the selection of MCAs for clinical use in tumour imaging.

### A study of the immunological effects of priming regimes

I.A. Fraser, V. Bowry, J. Collins & P.R.F. Bell

*Department of Surgery, University of Leicester, Leicester*

The use of a small "priming" dose of cytotoxic agents before major chemotherapy has recently been described (Hedley *et al.* (1978), *Lancet*, **2,** 966). This appears to reduce the toxicity of such therapy to the marrow and gastrointestinal tract, although its mechanism is not clear. Since immune depression is a serious consequence of most chemotherapy regimes, we have investigated the possibility of immunological protection afforded by such regimes using an animal model.

T cell function was measured in Sprague Dawley rats by two methods. *In vitro* PHA blastogenesis was studied by a whole blood technique and *in vivo* delayed type hypersensitivity (DTH) using oxazolone. One dose of a chemotherapeutic regime was administered to groups of animals as follows: cyclophosphamide, 5FU or CVMF (chlorambucil vincristine methotrexate and 5FU). Each regime produced clear depression of *in vitro* blastogenesis over the subsequent month and *in vivo* DTH response measured at 24 h. The effect of priming on this depression was investigated by a small dose of cyclophosphamide 5 days before regimes 1 and 3, and 5FU 5 days before regime 2. Groups of 20 animals simultaneously received one of the above regimes, half having been primed as above and half with saline as controls. No difference was observed in the subsequent depression of T cell function. In a further experiment using regime 1, the priming delay was varied from 0–14 days in 7 simultaneously studied groups of 10 animals. No difference was found in the subsequent immune depression of any group. It is considered that priming regimes are unlikely to confer protection to T lymphocyte function in patients receiving cancer chemotherapy.

### The incidence of leukaemia in B cell neoplasia

A.H.M. Abdul-Cader, P.R. Richardson, L. Walsh, I.C.M. MacLennan, M.J. Leyland, D.I. Gozzard, E.L. Jones & N.R. Ling

*East Birmingham Hospital; Department of Immunology, and Department of Histopathology, University of Birmingham*

Surface markers on blood lymphocytes were studied in 186 untreated patients with B cell neoplasia and correlated with the histopathological type of their disease using the Kiel classification. All of the 46 patients with diffuse lymphocytic lymphoma had B cell leukaemia, as defined by either $>90\%$ of B cells expressing surface membrane kappa or $>80\%$ expressing lambda. Of these, 39% had a total lymphocyte count of $<10^4\,\mu l^{-1}$. Twenty of the 65 patients with low grade follicle centre cell lymphoma had B cell leukaemia. On the other hand in myelomatosis only 1/35 had B cell leukaemia. In this case the cells were morphologically identified as plasma cells. In the lymphoplasmacytoid group the incidence of B cell leukaemia was 5/18 whilst in the high grade tumour group it was 3/22. This study has shown that B cell leukaemia is a common finding in low grade non-Hodgkin's lymphoma. It is less common in patients with high grade tumours and is not a feature of myelomatosis. In patients without B cell leukaemia an absolute reduction in blood B cell numbers was a common finding in all groups in our series. The clinical significance of B cell leukaemia or B lymphopenia associated with the various histopathological types remains to be determined. However, at this stage we can conclude that the assessment of light chain expression in blood lymphocytes in this group of diseases is an extremely useful diagnostic aid.

### Further advances in the management of malignant teratomas of the testis and other sites

E.S. Newlands, R.H.J. Begent, G.J.S. Rustin, D. Parker & K.D. Bagshawe

*Department of Medical Oncology, Charing Cross Hospital, Fulham Palace Road, London, W6 8RF*

Analysis of 100 male patients with metastatic malignant teratoma treated with sequential combination chemotherapy between 1977 and 1982 projects an overall survival of 72%. Following modification of the chemotherapy in 1979 (Newlands *et al.* (1980), *Br. J. Cancer*, **42**, 378) the survival in the last 69 patients entered in this study projects a survival of 80%. This chemotherapy, which alternates (a) vincristine, methotrexate, bleomycin, cis-platinum with (b) VP 16-213, actinomycin D and cyclophosphamide, has resulted in the salvage of patients even with very advanced disease. In complete remission are: 20/23 (87%) advanced abdominal disease, 20/24 (83%) with advanced lung disease, 11/19 (58%) with advanced abdominal and lung disease, 9/11 (82%) with liver metastases and 3/4 (75%) patients with brain metastases. Survival by primary site was: testis 72/87 (83%), retroperitoneum 3/7 (43%), mediastinum 3/4 (75%), unknown primary site 2/2 (100%). Multivariate analysis has identified two important adverse prognostic factors: the initial serum concentrations of human chorionic gonadotrophin (hCG) $>50\,000$ iu $l^{-1}$ and/or alpha-foetoprotein (AFP) $>500\,ugl^{-1}$; prior radiotherapy compromised prognosis ($P=0.017$). The most important determinant is the clinical concentration of hCG and/or AFP; in patients presenting with serum concentrations below the levels defined above the survival in 60 patients is 92%; and in those with higher concentrations the survival was 48% ($P = <0.001$).

**Response of human cervical tumours to radiotherapy determined by flow cytofluorometry**

J.E.D. Dyson, J.B. MacLaughlin, C.R. Surrey & C.A.F. Joslin

*University Department of Radiotherapy, Cookridge Hospital, Leeds LS16 6QB*

The technique of flow cytofluorometry enables the DNA and RNA content of individual cells, stained with a fluorescent dye, to be determined as they pass through a laser beam. By this means we are determining the content of heteroploid cells, and fraction of proliferative cells, in sequential biopsies of cervical tumours, taken at approximately weekly intervals, during a course of radiotherapy. Single cell suspensions are prepared from the tissue biopsies by disaggregation with collagenase, the cells stained stained with acridine orange plus ethidium bromide, then anlysed with an Ortho Diagnostics Systems 50 Cytofluorograf, with dedicated computer. All data are stored on disk for comparison with the histopathology determined on parallel biopsies, and ultimately to be compared with the long-term results of the therapy, and the progress of the patient. Results so far show that it is possible to monitor the elimination of heteroploid and proliferating cells during the course of treatment. Thus a rapid assessment may be made of the radiosensitivity of the tumour and the response to therapy. Continued accumulation of data should enable us to determine the extent to which flow cytofluorometric data may be utilised in the clinical management of cervical cancer, and individual patient prognosis.

**Adenocarcinoma of stomach, pancreas, biliary tract and unknown origin treated with 5-FU, adriamycin, mitomycin**

G. Sangster[1], D. Cunningham[2], A. Hutcheon[3], M. Soukop[2], S. Kaye[1], C. McArdle[2], K. Calman[1] & D. Carter[2]

[1]*Dept. of Clinical Oncology, Glasgow University,* [2]*Dept. of Oncology and Univ. Dept. of Surgery, Glasgow Royal Infirmary,* [3]*Woodend Gen. Hosp., Aberdeen*

Eighty-one patients (57 male, 24 female), mean age 55.8 y, received 5-Fluorouracil (F) twice monthly, Adriamycin (A) 3–4 weekly and Mitomycin-C (M) 6–8 weekly. Fifty-four had advanced gastric cancer (mean ECOG performance status (PS) 2.1), 16 adenocarcinoma of unknown primary site (ACUP) (mean PS 1.5) and 11 pancreatic or biliary cancer (mean PS 1.6). Six patients (3 gastric, 1 ACUP, 2 biliary) died within 3 weeks of starting FAM. The remaining 74 patients received at least 2 treatment cycles unless clear progression occurred after the first course and all are evaluable for response. In the gastric group, 32 (59.3%) patients showed disease progression (median survival (m.s.) 4 months), 6 (11.1%) were stable (m.s. 8.5 months), 10 (18.5%) had a partial response (PR) (m.s. 14.5 months) and 3 (5.6%) a complete response (CR) (m.s. 11 months). All patients in the pancreatic and biliary group showed progression (m.s. 3 months). The ACUP group included one patient with PR in a retroperitoneal mass, becoming CR with continued treatment at 6 months, the response being maintained at 18 months; 3 patients were stable and the remaining 12 with ACUP showed progression (m.s. 6 months). Of the 13 responders in the gastric group, 11 showed a response at the primary site while only 1 response was seen in a total of 15 patients with bulk liver disease. Toxicity was not severe, the major manifestation being haematological (mean nadir WBC $2.5 \times 10^9 l^{-1}$, mean nadir platelet count $90 \times 10^9 l^{-1}$). Alopecia was common and gastrointestinal toxicity (nausea and vomiting) was mild (mean grade 1.5). Overall the response to FAM chemotherapy was disappointing in this unselected patient population, suggesting that alternative drug therapy should be investigated as first line treatment for these forms of malignancy.

**Recombinant human interferon in advanced breast cancer**

H. Smedley, M. Katrak, K. Sikora & T. Wheeler

*Ludwig Institute for Cancer Research, Cambridge and Department of Radiotherapy, Addenbrookes Hospital, Cambridge*

The gene coding for human leucocyte A interferon (IFN-$\alpha$) has recently been isolated and cloned. We have used a highly purified preparation of recombinant IFN-$\alpha$ to study the efficacy and safety of this preparation in patients with advanced breast cancer. Twelve patients have received treatment with either 50 million units $m^{-2}$ i.m. three times weekly or 20 million units $m^{-2}$ i.m. daily for up to 12 weeks. A predictable pattern of side-effects has emerged, and a previously undescribed syndrome of central nervous system toxicity with abnormal EEG findings has been identified. This appears to be dose-related and reversible, but has been dose limiting in 4 patients. Five patients have had objective regression of tumour, but only 2 have met the UICC partial response criteria. Symptomatic improvement has been noted by 6 patients. Recombinant IFN-$\alpha$ clearly has some biological activity in patients with breast cancer and the trial will continue to assess its effects in a large series of patients.

### Human tumour metastasis: Studies on patients with malignant ascites treated with peritoneo-venous shunts

D. Tarin[1], A.C. Vass[2], R.G. Souter[3], J.E. Price[1] & B. Roach[1]

[1]*Nuffield Dept. of Pathology, John Radcliffe Hospital (Oxford Univ.),* [2]*Dept. of Obstet. & Gynaecol., Wycombe General Hospital, High Wycombe, Bucks.,* [3]*Nuffield Dept. of Surgery, John Radcliffe Hospital, Oxford*

The recent introduction of peritoneo-venous shunting for treatment of intractable ascites due to inoperable cancer has presented a unique opportunity for observing factors affecting metastatic spread of tumours in man. Since the shunt is inserted solely for the relief of pain and discomfort and investigative procedures are confined to ones required for good routine clinical management, the studies are ethically impeccable. We present observations on nine patients with primary tumours from a variety of sites, the majority being ovarian. The minimum time of survival was 2 months and the maximum 3 years. Cells from the ascitic fluid and from the blood were cultured, sometimes on several occasions, and autopsies were performed on eight of the patients. In some individuals no metastatic tumours formed in any organs, despite the fact that the tumour cells in the ascitic fluid were clearly viable and capable of forming numerous seedling colonies on the visceral and parietal peritoneum. Some of these patients had survived up to 3 years after insertion of the shunt and direct evidence of its patency was provided by clinical observation, culture of cells resembling tumour cells from the blood and presence of latent tumour cells with indisputable markers in many organs. Conversely, some patients had widely disseminated seedling tumours of uniform size in many organs within 2 months of shunt insertion. In none of the patients was any cellular immune response observed to deposits or latent tumour cells. The manner of tumour cell dissemination in these patients is the direct human counterpart of that used in our studies on metastatic colonisation potential of cells from spontaneous murine mammary tumours. The findings in the 2 species are so similar that we tentatively conclude that at least some of the underlying mechanisms in metastasis are common to both.

### Collagenase secretion and spread of human tumours

D.J. Ogilvie[1], A.C. Vass[2], E.G. Lee[3] & D. Tarin[1]

[1]*Nuffield Department of Pathology, (University of Oxford), John Ratcliffe Hospital, Oxford;* [2]*Department of Obstetrics & Gynaecology, Wycombe General Hospital, High Wycombe, Bucks;* [3]*Consultant Surgeon, John Radcliffe Hospital, Oxford*

There is a growing body of evidence that disruption of connective tissue is an essential feature of invasion and metastasis. In particular, destruction of collagen fibres has been demonstrated in the forefront of advancing tumours by electron microscopy (Tarin (1967), *Int. J. Cancer*, **2**, 195). *In vitro* production of collagenase, which is so far the only identified enzyme capable of such activity under physiological conditions, has been correlated with "metastatic" colonisation potential (Tarin *et al.* (1982), *Br. J. Cancer*, **46**, 266) for the MMTV-induced mouse mammary tumour and with temperature-dependent metastasis for the amphibian Lucké renal adenocarcinoma (Ogilvie *et al.*, in preparation). We have, therefore, studied certain human tumours to see whether collegenase has a role in invasion and metastasis and to assess its possible significance as a prognostic indicator. We have so far studied 60 breast samples (including 15 benign lesions) and 20 samples of gynaecological origin. These were all subjected to a primary explant culture regimen and harvested fluids were assayed for collagenolytic activity against [$^{14}$C]-radiolabelled, soluble, type I collagen. The viability of cultured material was verified by glucose uptake. The majority of breast tumours produce an enzyme, satisfying the criteria of a "true" vertebrate collagenase, which is mostly in a latent, trypsin activatable form. Collagenase output is generally high for benign tumours such as fibroadenomas and low for benign cystic disease. For malignant tumours, however, there is a wide quantitative spectrum of collagenase elaboration which shows *no* correlation with histological type. The question of whether these findings are of prognostic value is currently being evaluated in clinical trials.

**Levonantradol (LN) in the treatment of chemotherapy induced nausea and vomiting, refractory to conventional anti-emetics**

R. Stuart-Harris, C. Mooney & I.E. Smith

*Royal Marsden Hospital, London*

LN, a synthetic agent structurally related to delta 9 tetrahydrocannabinol, is suggested in preliminary reports as having anti-emetic activity in patients receiving cancer chemotherapy. In an open Phase II study the anti-emetic effect and toxicity of LN were assessed in patients in whom chemotherapy induced nausea and vomiting had proved intractable to conventional anti-emetics. Twenty-two patients (15 female, 7 male; age range 20–70 y) were treated with LN in an initial dose of 0.5 mg i.m. 4 hourly, starting one hour before chemotherapy and increasing if necessary to a maximum of 1.5 mg. One patient was not assessable for response as he refused further LN after one dose because of side effects. Of the 21 patients assessable for response, 2 (9%) had complete relief of nausea and vomiting, 7 (33%) good relief, 4 (19%) slight relief and 8 (38%) no relief. Side effects were reported by all patients and were worse with LN than with conventional anti-emetics ($P<0.001$). Side effects were drowsiness in 13 patients (59%), dizziness in 11 (50%), thought disturbance in 7 (31%), pain at injection site in 5 (22%) and dry mouth in 3 (13%). One patient developed severe but self limiting drowsiness with disorientation after a total dose of 7 mg in 36 h. Nine patients (40%) had side effects severe enough to reduce the dose or stop treatment. In conclusion, LN is an effective anti-emetic in approximately 50% of patients resistant to conventional anti-emetics; however its usefulness is likely to be restricted by side effects which are common and frequently unpleasant.

**Prospective cross over double-blind comparison of MST continus 30 mg and morphine sulphate solution cancer patients with pain**

J. Welsh[1], J.F.B. Stuart[1,2], T. Habeshaw[3], P. Billiaert[1], S.B. Kaye[3] & K.C. Calman[1]

[1]*Department of Oncology, University of Glasgow,* [2]*Department of Pharmaceutics, University of Strathclyde,* [3]*Glasgow Institute of Radiotherapeutics & Oncology*

The aim of this study was to compare the efficacy of 2 oral morphine preparations in the relief of severe and intractable pain. Fifteen patients, median age 56 y, completed the trial. Stabilisation on MST continus 30 mg (MST-3) tablets was achieved over a one week period. Patients were randomised to receive MST-3 active + Morphine sulphate solution (MSS) placebo or the reverse regime. After 7 days treatment the alternate regime was administered. Pain was assessed at intervals during the 3-week period by means of a visual analogue scale completed by the patients. Approximate duration of sleep and both incidence and intensity of side effects were recorded. Patients were permitted additional analgestics if break-through pain occurred. The results show MST-3 is an effective analgesic. There appears to be no difference in day or night pain control between MST-3 and MSS given in equivalent doses. There was no significant difference in the hours slept when taking the elixir or tablets. The percentage of patients developing side effects on the MST-3 and MSS was similar. The side effects were as expected, namely drowsiness, constipation, nausea, dizziness and vomiting. There was no significant difference in intensity of the side effects recorded ($P>0.5$). In summary MST-3 appears to have similar analgesic properties to that of equivalent doses of MSS. The side effects and intensity of these are similar for the two formulations. The advantage of the MST-3 preparation appears to be its twice daily regime and the convenience its formulation offers in terms of carriage by the patient.

**The effect of chemotherapy on saliva and oral micro-organisms**

B.E. Main[1], K.C. Calman[2], M.M. Ferguson[1], S.B. Kaye[2], T.W. MacFarlane[1], R.J. Mairs[1], L.P. Samaranayake[1], J. Welsh[2] & J. Willcox[2]

[1]*Department of Oral Medicine and Pathology, and* [2]*Department of Oncology, University of Glasgow*

Oral complications of chemotherapy result from direct mucosal damage or, indirectly, as a consequence of immunosuppression. Clinically there is also thought to be xerostomia and impaired taste. The aims of this study were to examine the effects of chemotherapy on saliva volume and composition (amylase, IgA and lysozyme). In addition, changes in the oral microbial flora were investigated. In the first study, healthy controls were compared to patients receiving chemotherapy for malignancies. This indicated that there were differences between the two groups. However, there were limitations on interpretation and a second longitudinal study was initiated. Twenty patients, median age 46 y, were assessed before starting chemotherapy and then again at one month and at three months of treatment. The 10-min salivary volume, amylase and IgA all fell significantly over the 3-month period. Lysozyme did not alter. The amount of candida isolated from saliva increased with chemotherapy and there was a correlation between salivary volume and candida score. A range of other potentially pathogenic bacteria were isolated from several of the patients, but not controls. The conclusion is that chemotherapy results in a decreased salivary flow, a lowering of salivary amylase and IgA, and an increase in opportunistic pathogens.

**Reduced erythropoietin levels as a cause of the anaemia of chronic disorders in patients with malignant disease**

R. Cox, T. Musial, S. Stafford & O.H.B. Gyde

*Departments of Surgery & Haematology, East Birmingham Hospital, Birmingham, B9 5ST*

The Anaemia of Chronic Disorder (ACD) is commonly associated with neoplastic (NG) disease. Investigation of Erythropoietin (Ep) levels using whole animal assays has given variable and conflicting results in this condition. In the present study Ep levels were measured using the Foetal Mouse Liver Assay in 40 patients with NG disease (33 small cell cancer of bronchus, 7 other NG) with a range of haemoglobin (Hb) levels and 19 normal controls. Anaemia and iron status were determined using full blood count, serum iron, transferrin and ferritin levels and bone marrow cytochemistry. Twelve patients had Hb $<11\,g\,dl^{-1}$ and the features of ACD. Thirteen other patients with NG had Hb $>12.5\,g\,dl^{-1}$ with some features of ACD but no evidence of a specific deficiency or marrow infiltration. Mean Ep level for the patients with Hb $<11\,g\,dl^{-1}$ and ACD was $0.22\,iu\,ml^{-1}$ which was significantly lower than for the controls—$0.31\,iu\,ml^{-1}$ ($P<0.02$). The patients with NG and Hb $>12.5\,g\,dl^{-1}$ had a mean Ep level of $0.26\,iu\,ml^{-1}$ These data support the concept that lack of an appropriate Ep response to a fall in Hb is one factor in the genesis of ACD of malignancy.

**Results of chronic lymphocytic and other leukaemias in a four-day tumour chemosensitivity assay**

A.G. Bosanquet, M.C. Bird & E.D. Gilby

*Royal United Hospital, Combe Park, Bath, Avon*

We are investigating the use of a relatively rapid and simple tumour cell assay as a means of predicting tumour response to cytotoxic drugs. The whole white cell fraction from blood or bone marrow is isolated by centrifuging over $1.090\,g\,ml^{-1}$ Ficoll/Isopaque and washed. The cells are set up at $2.5\times10^5$/tube in RPMI 1640 medium with 10% foetal calf serum. Drugs are added for either 1 h or continuously and the cells are incubated for 4 days at 37°C. A solution of a vital dye (fast green FCF) is added to stain the dead cells and they are cytocentrifuged. The live cells are counterstained with a modified haematoxylin/eosin or Romanowsky stain. Tumour cells are identifiable and are counted as a percentage of all cells. For drug-treated samples, the percent live tumour cells is calculated and expressed as a percentage of that in control samples (%TCV). Optimal drug concentrations for use *in vitro* have been determined for chlorambucil ($2\,\mu g\,ml^{-1}$), 4-hydroperoxycyclophosphamide ($2\,\mu g\,ml^{-1}$), vincristine ($0.1\,\mu g\,ml^{-1}$) and prednisolone ($0.5\,\mu g\,ml^{-1}$). Other drugs are also under investigation. From 20–200 control and drug-treated tubes are set up from each sample received and the number of cells has never been a limiting factor. Thirty-seven of 45 tests (82%) undertaken with CLL have been technically successful. From these, 11 assay results could be correlated with *in vivo* response. Two were sensitive both *in vitro* and *in vivo* and nine were resistant *in vitro* and *in vivo*.

**Simulation of genetic effects in white rats perinatally exposed to *fusarium* mycotoxins**

R. Schoental

*Dept. Pathology, Veterinary College, University of London, London NW1 0TU*

The great susceptibility of rapidly growing tissues to carcinogens is well documented. The differential susceptibility is often reflected in the striking differences between the effects of various xenobiotics on pregnant or lactating rats, and on their offspring. The latter may become affected by exposing the mother to doses of noxious agents, which leave the adult unscathed, as was first demonstrated using the hepatotoxic and hepatocarcinogenic pyrrolizidine alkaloids. In continuation of these studies, other substances were used which include the carcinogenic secondary metabolites of *Fusarium*. When relatively large doses of the oestrogenic zearalenone were given, either singly or combined with the trichothecene, T-2 toxin, to pregnant and/or lactating white rats, various tumours, benign and malignant, were found both in the treated mother-rat, and also in the offspring, male and female. The occurrence of endometrial neoplasms in mother and daughter rats appears to simulate familial tumours due to genetic factors.

### A new tumour marker system using primary cultures of rat hepatocytes

I. Hayashi & B. Carr

*Departments of Cytogenetics & Medical Oncology, City of Hope National Medical Center, Duarte, California 91010, U.S.A.*

Primary monolayer cultures of adult rat hepatocytes do not conventionally proliferate. However, when hepatocytes from male F344 rats, 200–230 g wt, were cultured in the presence of the 3 hormones (3H) glucagon $0.2\,\mu g\,ml^{-1}$, insulin $0.2\,\mu g\,ml^{-1}$, and EGF $10\,ng\,ml^{-1}$, DNA synthesis (DNA-S) occurred with a peak at 72–96 h after plating. The extent of DNA-S was cell density dependent, exhibiting a peak at $3 \times 10^5$ cells/35 mm diameter culture dish. Substratum was also important, since gelatin coating of the dishes induced greater DNA-S than fibronectin or plastic. Both glucagon and EGF displayed a linear dose response which then plateaued. However, insulin had a more complex response, with inhibition at higher doses. A large number of other hormones were tested, but added little to the above, except hydrocortisone which caused an inhibition of DNA-S. The effects of normal rat serum (NRS), calf serum, and normal human serum were tested in this system at 0.1–20%. All elicited DNA-S at 0.1–1.0% similar to that produced by 3H alone. However, above 3%, the only observable effect was an inhibition of DNA-S compared to 3H alone. Human plasma was similar to human serum, but human platelet-derived growth factor consistently inhibited DNA-S at $1.25\text{–}10\,\mu g\,ml^{-1}$. No difference was observed when NRS was compared to serum from rats containing regenerating livers at 1–36 h after partial hepatectomy. However, the serum from rats which were fed the hepatocarcinogen 2-AAF induced a 4-fold increase in the DNA-S compared to NRS, starting at 1 week after carcinogen administration. The $105{,}000 \times g$ supernatant of 2-AAF livers also increased the level of DNA-S in the test hepatocytes compared to the similar extract from normal liver. These presumptive tumour growth factor(s) are now being further characterized.

### Manipulation of hepatic γ-glutamyl transpeptidase activity in control and $AFB_1$-fed animals to study its role in detoxification of carcinogens

M. Manson, G.E. Neal & E. Moss

*MRC Toxicology Unit, Carshalton, Surrey, SM5 4EF*

Gamma glutamyl transpeptidase (γGT) found in the bile duct epithelium in normal adult rat liver, appears in foci of hepatocytes (so-called preneoplastic lesions) in animals exposed to one or more of a wide range of carcinogens. Among the proposed functions of γGT in normal liver are involvement in maintenance of intracellular glutathione levels and also in breakdown of glutathione conjugates in the first stage of mercapturic acid formation. The function of the enzyme in preneoplastic foci is poorly understood, although such hepatocytes may be more resistant to cytotoxic effects of the carcinogen. In the present study bile was collected from control adult male Fischer 344 rats and from animals fed a diet containing 4ppm $AFB_1$ for at least 10 weeks. Fifteen mins before cannulation some animals received 11 mg AT125 i.p. and immediately prior to bile collection some animals from each group received i.v. $5\,\mu M\,kg^{-1}$ $AFB_1$. γGT activity was examined histochemically in liver and quantitatively in liver and bile. The enzyme was present in the bile duct epithelium in control animals and also in numerous foci in fed animals (5–10x control levels). γGT activity could be measured in bile from control and fed animals but no particular pattern emerged. In animals treated with AT 125, a specific inhibitor of γGT, no enzyme activity was visible histochemically in any lobe of the liver and no activity could be measured in liver homogenates or in bile. γGT activity was also measured in female rats (which are more resistant to toxic and carcinogenic effects of $AFB_1$) and found on average to be $2\times$ that of males, present in hepatocytes as well as bile ducts. These animals were then used to study the effect of altered γGT activity, particularly in hepatocytes, on the pattern of $AFB_1$-conjugates excreted into bile following a single i.v. dose of the carcinogen as described in the next abstract.

### The effect of manipulation of γGT on the biliary excretion of aflatoxin $B_1$-conjugates

E.J. Moss, G.E. Neal & M. Manson

*MRC Toxicology Unit, Carshalton, Surrey SM5 4EF*

A glutathione conjugate of the reactive epoxide intermediate of the carcinogen aflatoxin $B_1$ ($AFB_1$), has been prepared *in vitro*. $^1$H n.m.r. and mass spectrometry identified the conjugate as the 8,9-dihydro-8(S-glutathionyl)-9-hydroxy aflatoxin $B_1$ ($AFB_1$-GSH). This conjugate was shown to be a substrate for $\gamma$-glutamyl transpeptidase ($\gamma$GT) *in vitro* demonstrating the presence of a free, transferable $\gamma$-glutamyl moiety. Incubations with a hepatoma cell line rich in GGT resulted in the disappearance of $AFB_1$-GSH on HPLC analysis and the appearance of a new material, with a longer retention time, identified as the cysteinyl-glycl conjugate of $AFB_1$ ($AFB_1$-Cys Gly). This transformation was inhibited by the specific GGT inhibitor, AT 125. *In vivo,* following i.v. injection of $AFB_1$ to control male Fischer 344 rats ($5\,\mu M\,kg^{-1}$), $AFB_1$-GSH was detected in the bile as a major $AFB_1$ metabolite over the first 2h. Although the conjugate passes through an area of the liver rich in GGT the rate of conversion of $AFB_1$-GSH to $AFB_1$-Cys Gly at this dose level was very low ($AFB_1$-GSH: $AFB_1$-Cys Gly $\approx$ 10:1). However, in animals which had been fed 4 ppm $AFB_1$ for a 12-week period prior to the experiment, the pattern of biliary excretion was dramatically altered. The $AFB_1$-Cys Gly levels were elevated such that $AFB_1$-GSH:$AFB_1$-Cys Gly ratios of 1:1 were recorded. Administration of AT 125 to the animals immediately prior to the experiment inhibited conversion to $AFB_1$-Cys Gly and the amount detected was approximately $\leqslant$ to levels in control animals ($AFB_1$-GSH:$AFB_1$-Cys Gly $\approx$ 40:1). These results show the influence of altered GGT levels on the subsequent metabolism of carcinogens and provide information on the site of metabolism of carcinogens in the preneoplastic liver.

### Investigations of mechanisms responsible for alterations in drug sensitivities occurring after long-term radiation exposure

A.S. Bellamy & B.T. Hill

*Laboratory of Cellular Chemotherapy, Imperial Cancer Research Fund, London, WC2A 3PX*

Alterations in response to chemotherapeutic agents after long-term radiation (DXR) exposure of mammalian tumour cell lines *in vitro,* have been reported previously (Bellamy & Hill, (1982), *Br. J. Cancer,* **45,** 640). For DXR-pretreated HN-1 cells (derived from a squamous cell carcinoma of the tongue), the most marked changes noted were significantly reduced sensitivities to 24h exposures to vincristine or VP16-213, and a significantly increased sensitivity to 5-fluorouracil (24h exposure), compared with responses to untreated parent cells. Studies of cellular [$^3$H]-vincristine transport showed no difference in drug uptake by radiation-pretreated and parent HN-1 cells, despite the marked resistance of the radiation-treated line to vincristine. Alkaline sucrose gradient sedimentation profiles showed evidence of single strand breaks in DNA after a 24h exposure to VP16-213. However, these were present in both parent and radiation-pretreated cells, which implies that such lesions may not be related to the differential cytotoxic responses observed. Cellular levels of two of the enzymes involved in 5-fluorouracil metabolism were determined. Thymidine kinase activity was comparable in both untreated and radiation pretreated cells. The activity of thymidylate synthetase, however, was significantly decreased in radiation-pretreated cells. This may be associated with the increased sensitivity of these cells to 5-fluorouracil. If confirmed, this finding of specific DXR-induced cellular changes, associated with altered drug responses has major implications for the combined modality treatment of human cancers.

### Studies on fluorescent probes for hypoxic cells

A.C. Begg, N.J. McNally, R.J. Hodgkiss & P. Wardman

*CRC Gray Laboratory, Mount Vernon Hospital, Northwood, Middlesex HA6 2RN*

Certain nitroimidazoles have been found to undergo nitroreduction with subsequent binding in hypoxic cells, but not in oxic cells. We have attempted to utilize this differential by developing compounds which become fluorescent on nitroreduction, a process which occurs only in hypoxic cells. Such compounds could, in principle, be used to quantitate and separate hypoxic cells in tumours using a flow cytometer/cell sorter. To date, two compounds have been studied, viz. Eosin B and nitroacridine. V79-379A cells grown in suspension culture were incubated with the drug in air and hypoxia and tested for fluorescence using an Ortho Cytofluorograph, System 50H. Fluorescence histograms were obtained for cells incubated with the compounds at different concentrations for various times. With $500\,\mu M$ Eosin B the mean fluorescence of hypoxic cells was greater than that for oxic cells for incubation times up to 3h; however, there was considerable overlap of the fluorescence histograms. With nitroacridine, almost complete separation of the fluorescence histograms for oxic and hypoxic cells was obtained and at concentrations showing minimal cytotoxicity. In multicellular spheroids of V79-171 B cells incubated with nitroacridine and grown in medium gassed with air, a proportion of cells were fluorescent indicating the presence of hypoxic cells. The proportion depended upon the size of the spheroids and the frequency of changing their growth medium. A comparison of the fluorescent fraction with the radiobiologically determined hypoxic fraction will be made.

**Antimetastatic effect of cimetidine against the $C_3H$ mouse mammary adenocarcinoma**

M. Penhaligon, J. Anthoons[1], D. Pilkington, R.A. Wolstencroft[1] & A.H.W. Nias

*Richard Dimbleby Department of Cancer Research and [1]Immunology Department, St. Thomas's Hospital Medical School, London SE1 7EH*

There are no effective antimetastatic agents that are not also highly toxic. Osband *et al.* (1981), *Lancet*, **i**, 636, found that Cimetidine prolonged survival and slowed metastatic development in mice bearing the Lewis lung carcinoma. They suggested this was due to blockade of the suppressor cell system by Cimetidine, a histamine-2 receptor antagonist. Using the $C_3H$ mouse mammary adenocarcinoma we have examined the effect of Cimetidine on survival, metastasis development and the response of spleen lymphocytes to mitogens. Lung metastases were produced by implanting tumours s.c. on the back and allowing them to grow to a mean diameter of 5.3 mm when they were ablated with 90 Gy x-irradiation. Metastases became detectable after about 40 days. Cimetidine was given in the drinking water ($10\,mg\,ml^{-1}$) from the time of irradiation until the end of the experiment. Cimetidine reduced the number of mice dying from metastases from 47% (controls) to 24%. Cimetidine also slowed metastatic development. The response, in terms of thymidine incorporation, of spleen lymphocytes from Cimetidine treated and control mice to the T-cell mitogen Phytohaemagglutinin and the B-cell mitogen Lipopolysaccharide was depressed or enhanced according to mitogen dose and time of assay during the course of Cimetidine treatment and metastatic development. The mechanism of action of Cimetidine's antimetastatic effect remains unclear.

**Site-specific growth of rat tumours**

N. Willmott, J. Newton[1] & K.C. Calman

*Department of Oncology, University of Glasgow, and [1]Department of Haematology, Western Infirmary, Glasgow*

Whilst much effort has been directed towards quantifying tumour growth in the lungs of experimental animals, few studies have compared growth in pulmonary tissue with growth at other sites. Consequently, in these studies we have examined the growth of tumours Mc7, Mc40A, Walker 256 and Sp 24 following i.v. and s.c. injection into Nottingham Wistar rats, using a method that is objective, amenable to statistical analysis and accurately reflects numbers of clonogenic tumour cells. The technique involved recording incidence of tumour growth following i.v. and s.c. injection of graded doses of cells from each tumour and calculation of TD-50 values. It was found that Mc7 and Sp 24 cells were significantly more tumourigenic following i.v. injection than s.c. injection, whereas the converse was true of Walker 256 cells. Tumourigenicity of Mc40A cells was not significantly different following i.v. and s.c. injection. Growth of all tumours after i.v. injection was confined to the lungs. Experiments with radiolabelled tumour cells injected i.v. revealed that those tumours exhibiting increased tumourigenicity following i.v. injection, relative to s.c. injection, appeared better able to survive the attrition generally seen when tumour cell suspensions are injected into the blood stream. Further experiments indicated that association of tumour cells, either with platelets or other tumour cells, was correlated with higher tumourigenicity following i.v. injection relative to s.c. injection.

**Spontaneous and experimental metastasis of primary mammary tumours: Back titration of cell shedding**

J.E. Price & D. Tarin

*Nuffield Department of Pathology (University of Oxford), John Radcliffe Hospital, Oxford*

Spontaneous mammary tumours of mice differ in metastatic colonisation potential, some producing many pulmonary deposits (high colonisation potential—HCP), others producing few or none (low colonisation potential—LCP) after intravenous inoculation (Tarin & Price (1979), *Br. J. Cancer*, **39**, 740). Separate tumours from a single mouse can have independent colonisation potentials, indicating that this is a property of the neoplasm rather than the host. Serial dilution experiments show that HCP tumour cells still produce large numbers of lung deposits when the dose is reduced to $10^3$ or $10^4$ viable cells. The standard assay dose of $5 \times 10^5$ cells is, therefore, 50 times in excess of that required for the definitive colonisation grade. Differences between HCP and LCP tumours remain stable over a wide range of doses, although are not absolute; some LCP tumours can colonise the lungs heavily, but only at doses 250 times greater than that required for HCP tumours to give a similar result. The incidence of spontaneous metastasis in a sample of 200 primary mammary tumours in C3H/$A^{vy}$ mice was found to be 33%. When inoculated i.v., cells from some of these metastatic tumours had HCP and others LCP and the degree of spontaneous metastasis did not always coincide with the i.v. result. From the assayed colonisation potential of a tumour, the degree of spontaneous metastasis and knowledge of dose response, one can back-titrate the degree of cell shedding from a particular tumour. For example, tumours which were of LCP by i.v. assay but had produced numerous pulmonary deposits spontaneously must have shed enormous numbers of cells into the blood. Conversely, those tumours of HCP which produced few spontaneous metastases must have shed exceedingly few cells (Price *et al.* (1982), *Invasion & Metastasis*, **2** (in press).

### Degradation of basement membrane collagens by disseminating tumours in three vertebrate species

S.E. Shields, D.J. Ogilvie, R.G. McKinnell & D. Tarin

*Nuffield Department of Pathology (University of Oxford), John Radcliffe Hospital, Oxford*

Observations on tumour invasion and metastasis have demonstrated extensive destruction of the adjacent connective tissues and basement membranes. The deduction that the breakdown of collagen is caused by the local extracellular release of neutral proteases is supported by studies demonstrating the secretion of a specific collagenase active against type I (fibrous) collagen by murine mammary tumours (Tarin *et al.* (1982), *Br. J. Cancer*, **46**, 266). However, it has been found that collagen types IV and V, which are structurally distinct from collagen type I, are resistant to this previously isolated collagenase. We have, therefore, looked for secretion of collagenases capable of digesting these components of basement membranes.

Types IV and V collagen were obtained from human placentae by pepsin digestion followed by salt fractionation. Supernatants from explant cultures of murine mammary tumours, human breast and endometrial carcinomas, and amphibian renal tumours were tested for protease activity against these collagenous substrates. Breakdown of substrates and appearance of specific reaction products were displayed by polyacrylamide gel electrophoresis. Confirmation that substrate cleavage was due specifically to collagenase activity was obtained by its inhibition in the presence of EDTA. The supernatants from all three species studied were capable of digesting type IV collagen; one human breast tumour supernatant also digested type V collagen. The degree of substrate cleavage differed between tumours and activity against types IV and V collagen varied independently indicating the presence of two separate enzymes. These findings support the interpretation that the destruction of collagen is an important and general mechanism underlying tumour invasion and metastasis in several species.

### Changes in tumour cell kinetics with distance from a blood vessel

B. Jones & R.S. Camplejohn

*Richard Dimbleby Department of Cancer Research, St. Thomas's Hospital Medical School, London SE1. 7EH*

Cell kinetic variations within a tumour due to a variety of factors including vascular proximity may have important implications for cancer therapy. In this study the stathmokinetic effect of Vincristine (VCR) was used to determine cell birth rates ($k_B$) at increasing distances from central blood vessels occurring in corded areas of transplantable $C_3H$ mouse mammary tumours. This method has many advantages over tritiated thymidine techniques used by other authors in similar situations. For example, the length of time required to perform fraction labelled mitosis or continuous labelling experiments is such that migration of tumour cells may seriously complicate the results. In contrast a metaphase arrest experiment can be completed within 3 hours, thus minimizing the effect of cell migration. This method, when carefully performed, yields a direct estimate of $k_B$ and also provides 95% confidence limits for each result. $k_B$ was estimated by counting 1000 cells in each of the following zones: A (proximal to vasculature), B (intermediate) and C

(furthest from vasculature and adjacent to necrosis). A progressive reduction in native mitotic index, cell birth rates and calculated growth fraction was seen from zone A to zone C. Mean $k_B$ expressed as new cells/1000 cells/hour with 95% confidence limits in brackets for zone A, B and C respectively were: 29.9 (19.1–40.7); 18.7 (9.6–27.8); 4.8 (1.9–7.7). The estimated duration of mitosis remained fairly constant in each zone (A—47 min, B—44 min and C—56 min). No ana- or telophases were seen in zone C, demonstrating that diffusion of VCR was sufficient to cause complete metaphase arrest. These results demonstrate a clear inverse relationship between cell proliferation and distance from a blood vessel.

**Oestrogens, malignant disease and folate metabolism**

J.A. Blair, P.A. Barford, A.E. Pheasant, A.E. Guest, C. Eggar & G.D. Oates

*Department of Chemistry, University of Aston in Birmingham, Birmingham B4 7ET and The General Hospital, Steelhouse Lane, Birmingham, B4 6NH*

In man and the rat with malignant disease folate metabolism alters so as to reduce folate breakdown by scission and to increase the proportion of folates present as 10-formyltetrahydrofolate derivatives. Thus the concentration of the folate coenzymes crucial for the synthesis of purines and thymidylate for RNA and DNA increases. In oestrogen-sensitive human breast tumours but not in human gut tumours dihydropteridine reductase activity, an enzyme reducing folate scission, is significantly increased compared to adjacent apparently normal tissue. Oral administration of diethylstilboestrol to female rats significantly reduces folate breakdown and greatly increases the proportion of 10-formyltetrahydrofolate. Thus oestrogen appears to alter folate metabolism to a pattern required for proliferating tissue. Anti-oestrogen therapy of breast tumours may act by altering folate metabolism.